# Genomic Insights into Inbreeding, Population Structure, and Selection Signatures in Salem Black and Global Goat Breeds

**DOI:** 10.3390/ani16193123

**Published:** 2026-10-05

**Authors:** Oludayo Michael Akinsola, Malarmathi Muthusamy, Arun Kumar Chinnasamy, Chitra Ramasamy, Pritam Pal, Saravanan Ramasamy, Mani Jeyakumar, Imaben Grace Opaluwa-Kuzayed, Aranganoor Kannan Thiruvenkadan, Sunday Olusola Peters

**Affiliations:** 1 Department of Theriogenology and Production, Faculty of Veterinary Medicine, University of Jos, Jos 930103, Nigeria; akinsolao@unijos.edu.ng (O.M.A.); oimabengrace@yahoo.com (I.G.O.-K.); 2 Department of Animal Genetics and Breeding, Veterinary College and Research Institute, Tamil Nadu Veterinary and Animal Sciences University (TANUVAS), Namakkal 637002, Tamil Nadu, India; malarmathi.m@tanuvas.ac.in (M.M.); arunkumar19299@gmail.com (A.K.C.); chitra.r@tanuvas.ac.in (C.R.); jeyakumar.m@tanuvas.ac.in (M.J.); 3 Animal Genetics and Breeding Division, Indian Council of Agricultural Research (ICAR)-National Dairy Research Institute (NDRI), Karnal 132001, Haryana, India; pritamvet1@gmail.com; 4 Department of AH Statistics and Computer Applications, Veterinary College and Research Institute, Tamil Nadu Veterinary and Animal Sciences University (TANUVAS), Namakkal 637002, Tamil Nadu, India; saravanan.r@tanuvas.ac.in; 5 College of Poultry Production and Management, Tamil Nadu Veterinary and Animal Sciences University (TANUVAS), Hosur 635110, Tamil Nadu, India; 6 Department of Animal Science, Berry College, Mount Berry, GA 30149, USA

**Keywords:** indigenous goats, genomic inbreeding, runs of homozygosity (ROH), selection signatures, polygenic adaptation, population structure, effective population size, livestock conservation genetics

## Abstract

The Salem Black goat is an indigenous Indian breed valued for its premium Black leather and high twinning rate, yet its genomic diversity and adaptive potential remain unexplored. We characterised genomic diversity, population structure, demographic history and selection signatures using whole-genome sequence data from 11 Salem Black goats (7 unrelated individuals retained after kinship filtering) analysed alongside 157 individuals from five global breeds (Red Sokoto/Maradi, Tanzania Toggenburg, Omani Jabal Akhdar, Pakistani Kachan and Madagascar Diana), yielding a final dataset of 120 unrelated animals and 36,584 autosomal SNPs. Genomic inbreeding was estimated via runs of homozygosity; population structure was assessed using principal component analysis, ADMIXTURE, phylogenetic reconstruction, and genetic differentiation. Historical effective population size was inferred, and selection signatures were identified using the de-correlated composite of multiple signals (DCMS) approach. Salem Black goats showed the highest genomic inbreeding ($F_{ROH}$ = 0.41), lowest observed heterozygosity (9.6%), and smallest recent effective population size ($N_{e}\approx 15$), indicating severe recent inbreeding and genetic erosion. Population structure analyses identified Salem Black as a distinct lineage most closely related to Pakistani Kachan goats. Significant selection signals emerged only in Madagascar Diana, Omani Jabal Akhdar, and Tanzania Toggenburg goats, highlighting genes linked to heat tolerance, immunity, DNA repair, metabolism, and reproduction. These findings provide the first genomic characterization of Salem Black goats and identify their urgent conservation priority.

## Simple Summary

The Salem Black goat is an indigenous Indian breed valued for its premium Black leather, adaptability to harsh environments, and high twinning ability. Despite its economic importance, its genetic status has remained largely unexplored. Using genome-wide single nucleotide polymorphism (SNP) data, we evaluated genetic diversity, inbreeding levels, and adaptive signatures in Salem Black goats and compared them with global goat populations. Our findings reveal extremely high inbreeding, extensive long runs of homozygosity, and a critically small effective population size in Salem Black goats, indicating recent genetic erosion and heightened vulnerability. In contrast, several African and Middle Eastern breeds exhibited genomic signals associated with heat tolerance, immunity, metabolism, and reproduction, reflecting their adaptation to diverse and challenging environments. The absence of detectable selection signals in Salem Black goats is likely due to overwhelming genome-wide homozygosity rather than a lack of adaptive potential. These results highlight the urgent need for conservation and structured breeding strategies to preserve this unique genetic resource.

## 1. Introduction

India is home to the world’s second-largest goat population, comprising approximately 149 million animals [1], rendering goats an essential livelihood asset for marginal and landless smallholder farmers [2]. Among the 43 recognised indigenous goat breeds of India, the Salem Black is a geographically confined yet economically significant breed endemic to the Salem, Dharmapuri, Erode, Krishnagiri, and Namakkal districts of Tamil Nadu [3]. The Salem Black is distinguished for its glossy Black coat, elevated twinning rates, and superior Black hide prized by the leather industry [3,4]. Under extensive management conditions, the breed attains body weights of 20.71–48.64 kg [5]. Well-suited to a demanding hot semi-arid tropical climate marked by extreme heat, irregular precipitation, recurrent droughts, and sparse forage, the Salem Black goat is reported to possess robust adaptive attributes, such as heat tolerance, disease resistance, proficient water retention, and the capacity to subsist on low-grade fodder [6,7]. Notwithstanding these evident adaptations and its commercial value to southern India, the genetic underpinnings of these traits remain unexamined, with no comprehensive genomic analyses undertaken for this breed to date, despite uncontrolled breeding among smallholder farmers. Moreover, the dearth of pedigree documentation in village-based systems in India has hindered organised breeding, conservation, and enhancement initiatives. Prior microsatellite analyses of Tamil Nadu goat ecotypes, encompassing Salem Black, Kanni Adu, and Kodi Adu, indicated modest genetic differentiation [8], albeit constrained by limited sample sizes and sparse marker coverage. Recent genomic surveys of Indian goat breeds have documented disparate inbreeding levels ($F_{ROH}$ from 0.004 in Changthangi to 0.602 in Jharkhand Black) [9], yet omitted the Salem Black, underscoring the imperative for breed-tailored, high-resolution genomic profiling. Contemporary research has pinpointed selection signatures in selected Indian breeds under smallholder systems [9,10], though these frequently employed restricted marker sets and solitary statistical approaches, such as runs of homozygosity, may miss polygenic selective forces [9,11,12]. Studies using runs of homozygosity islands have identified selection sweeps in key genes governing cold adaptation and hair follicle development (*APOBEC1*, *DNAJC3*, *F2RL1*, *FGF9*) in pashmina breed; intramuscular fat deposition, meat tenderisation, lean meat production and carcass weight (*CDK4*, *ALOX15*, *CASP9*, *PRDM16*, *DVL1*) in meat breeds; while milk fat percentage and mammary gland development (*POLD1*, *NOTCH2*, *ARHGAP35*) in meat and dairy breeds. Progress in whole-genome resequencing (WGRS) now facilitates fine-scale assessment of inbreeding, demographic trajectories, population structure, and composite selection signals [13]. Employing composite methodologies for fine-scale evaluation of the Salem Black goat is crucial to elucidating local adaptation. Here, we combine multiple genomic inbreeding measures [14,15,16,17], reconstruct historical effective population sizes [18], and gauge population differentiation (F*_ST_*) alongside principal component analysis (PCA) to delineate the interplay between demographic dynamics and environmental selection [19,20,21,22,23]. We further augment single-population selection scans using a de-correlated composite of multiple signals (DCMS) framework [11,24]. This approach integrates five complementary statistics calculated in non-overlapping 500 kb windows: three haplotype-based measures (integrated haplotype score [iHS], number of segregating sites by length [nSL], and haplotype homozygosity [H12]) and two frequency-based measures (pooled heterozygosity [ZHp] and Tajima’s D) [25,26,27].

In this study, we present the first genome-wide sequence data for the Salem Black goat and place the breed within a global comparative framework by analyzing it alongside five genetically diverse goat populations from Africa, South Asia, the Middle East, and European-influenced lineages [28]. This design facilitates a comprehensive assessment of inbreeding patterns, genetic distinctiveness, admixture history, and genomic regions under positive selection in the Salem Black goat relative to global goat diversity. We hypothesize that owing to its restricted geographic distribution and the absence of formal pedigree management, the Salem Black goat will exhibit distinct population structure and elevated genomic inbreeding compared to more widely distributed reference populations. This study aims to: (i) characterise genomic diversity and inbreeding patterns in the Salem Black goat and global reference populations using ROH-based and other genomic inbreeding metrics, and (ii) identify candidate genomic regions under positive selection through DCMS and runs of homozygosity (ROH) selection-signature analyses.

## 2. Materials and Methods

### 2.1. Study Populations and Data Sources

The study combined newly generated whole-genome resequencing data from Salem Black goat with publicly available genotyping data from the AdaptMap project and additional breed (Oman Jabal Akhdar goat; [29]).

Salem Black goat resequencing: Eleven adult Salem Black goats (5 males and 6 females) were sampled from four geographically distinct locations in the north-western agro-climatic zone of Tamil Nadu, India (Mecheri, Kolli Hills, Bargur, and Livestock Farm Complex, Veterinary College and Research Institute, Namakkal), representing both traditional low-input and institutional management systems. All procedures were approved by the Institutional Animal Ethics Committee of Tamil Nadu Veterinary and Animal Sciences University. Genomic DNA was extracted from EDTA-anticoagulated jugular blood using the standard phenol–chloroform–isoamyl alcohol protocol [30]. DNA quality was verified by NanoDrop (Thermo Fisher Scientific, Waltham, MA, USA; A260/A280 and A260/A230 ratios) and 1% agarose gel electrophoresis. Whole-genome resequencing (150 bp paired-end) was performed on the Illumina NovaSeq 6000 platform (Illumina Inc., San Diego, CA, USA) by Oneomics Private Limited, Tiruchirappalli, Tamil Nadu, India. Raw data ranged from 3.16–31.44 Gb per sample with Q30 >96% and GC content 41–45%. Clean reads were aligned to the goat reference genome ARS1.2 (GCF_001704415.2) using BWA v0.7.17. Variant calling was performed with GATK HaplotypeCaller v4.4.2.1 and ANGSD v0.918. SNPs were filtered (QD > 2, missingness < 0.1, MAF > 0.05), yielding 40,376 high-quality SNPs across 11 individuals after sample quality control. All sequencing data are deposited in NCBI under BioProject PRJNA1302180 (BioSamples SAMN50465512–SAMN50465522; SRA SRR34954288–SRR34954298). Detailed per-sample sequencing statistics including raw data volume, Q30 scores, GC content, and mapping rates are provided in Supplementary Table S5. All animal procedures used in this study were conducted in accordance with institutional guidelines for the care and use of animals. Ethical approval for the genomic inbreeding study was obtained from the Institutional Animal Ethics Committee of VCRI, Namakkal, India, with approval number 07/VCRI-NKL/2025.

Public comparative dataset: Genotypic data for additional breeds were obtained from the AdaptMap project [28,29], comprising 157 individuals from five breeds: Oman Jabal Akhdar (OM_JAK: 72), Madagascar–Diana (MG_DIA: 17), Nigeria–Red Sokoto (NG_RSK: 21), Tanzania–Toggenburg (TZ_TOG: 23) and Pakistan Kachan (PK_KAC: 24). All individuals were genotyped on the goat 50K SNP array and mapped to the ARS1 reference genome.

### 2.2. Dataset Merging and Quality Control

The Salem Black resequencing SNPs and the public AdaptMap genotypes were merged in PLINK v2.0 [14], restricting the data to autosomal markers on chromosomes 1–29 (*PLINK* --chr-set 29 --autosome), resulting in 36,584 autosomal SNPs. Quality control steps were applied as follows: Individuals with >10% missing genotypes were removed (none failed). SNPs with call rate < 95%, MAF < 0.05, or Hardy–Weinberg exact *p* < 1 × 10^−6^ were excluded, retaining 35,857 SNPs. To minimise kinship bias, some individuals from each pair with PI_HAT > 0.25 were removed, yielding 120 unrelated individuals (OM_JAK: 43, MG_DIA: 13, IN_SAL: 7, NG_RSK: 21, TZ_TOG: 18, PK_KAC: 18). For analyses requiring approximately independent markers (PCA and ADMIXTURE), linkage disequilibrium (LD) pruning was performed (50-SNP window, 5-SNP step, r^2^ threshold = 0.2), resulting in a final pruned set of 23,639 SNPs (Table S6).

### 2.3. Runs of Homozygosity (ROH) and Genomic Inbreeding

ROH were identified using PLINK v1.9, guided by small-ruminant-oriented recommendations [31]. We set a minimum ROH length of 1000 kb (--homozyg-kb 1000) and allowed gaps of up to 1000 kb (--homozyg-gap 1000) to reduce artificial fragmentation in genomic regions with moderate SNP spacing. Each sliding window of SNPs could not contain any heterozygous calls (--homozyg-window-het 0) and could tolerate one missing call (--homozyg-window-missing 1). A density threshold of one SNP per 150 kb (i.e., --homozyg-density 150) helped maintain consistency in coverage. We applied breed-specific minimum SNP thresholds (i.e., --homozyg-snp) using the L-parameter approach to account for variation in local linkage disequilibrium. The final ROH segments were sorted into categories of 1–4, 4–6, 6–8 and ≥8 Mb length classes which broadly correspond to progressively more recent inbreeding events [32], though this classification is approximate and influenced by SNP density, detection parameters, and sample size.

Multiple genomic inbreeding coefficients were computed to capture different facets of autozygosity. $F_{ROH}$ was obtained as the proportion of total autosomal coverage in ROH, where the numerator is the total length of ROH per individual, and the denominator is the total genomic length covered by SNPs. The variance-standardized genomic relationship coefficient ($F_{GRM}$) was calculated using the --ibc function, allowing for the possibility of negative values if individuals exhibit higher heterozygosity than predicted by the reference allele frequencies [14,15]. $F_{HOM}$ measured deviations in homozygosity relative to Hardy–Weinberg expectations [14]. These metrics were collectively evaluated to reduce ambiguity that might arise from any single estimator [16,17]. Breed-specific means for ROH lengths and all inbreeding coefficients were compared by one-way analysis of variance, followed by Tukey–Kramer post hoc tests in the agricolae v1.3-7 R package [33].

We employed PLINK to calculate various genomic inbreeding coefficients to provide a multifaceted view of inbreeding. The genomic inbreeding coefficient ($F_{ROH}$) was calculated using the following formula [34]: $F_{ROH}$ = ∑L_ROH_/L_AUTO_, where ∑L_ROH_ represents the total length of all ROH in the genome of an individual, and L_AUTO_ is the length of the autosomal genome covered by SNPs in the analysis. Additionally, we calculated three different estimates of the genomic inbreeding coefficient for each breed: $F_{GRM}$ based on the variance-standardized relationship 1, $F_{HOM}$ estimated from the excess of homozygotes, and $F_{UNI}$ derived from the correlation between uniting gametes [15]. These coefficients $F_{GRM}$, $F_{HOM}$, and $F_{UNI}$ were computed using the --ibc flag in PLINK. The genomic inbreeding coefficient for each breed was determined by averaging these coefficients across all individuals within the breed.

To evaluate differences in ROH, genomic inbreeding metrics, and genome coverage among goat breeds, we conducted an analysis of variance (ANOVA) followed by a Tukey–Kramer post-hoc analysis using the aov function for ANOVA and HSD.test functions from the R package agricolae v1.3-7 [33]. This approach allowed us to identify significant disparities at *p* < 0.05, accommodating comparisons across groups with unequal sample sizes. To further analyse the data, we conducted a correlation analysis to examine the relationships between the distributions of ROH and the various inbreeding metrics. Employing the cor. test function in R v4.4.2, we calculated Pearson’s correlation coefficients and determined *p*-values at significance levels of 0.001, 0.01, and 0.05 to evaluate the statistical significance of the observed correlations. The correlation analysis was displayed using the R package corrplot v0.92 [35].

#### Detection of Runs of Homozygosity Islands

Detection of ROH islands was performed using PLINK to quantify ROH incidence, defined as the percentage of animals within a population having an SNP within an ROH segment. ROH incidences were visualised as Manhattan plots using R package qqman v0.1.9 [36]. ROH islands, indicative of positive selection, were defined based on SNPs exceeding a population-specific threshold. This threshold, derived from standard normal z-scores of ROH incidences, set a cutoff where the top 0.1% of SNPs with a *p*-value over 0.999 were considered significant [16,37]. A minimal incidence threshold of approximately 35% was required for an ROH to qualify as an island as an additional restriction, with the precise count taken as the nearest integer number of individuals given the discrete sample size of each breed.

### 2.4. Population Structure

The LD-pruned dataset of 23,639 SNPs (derived after applying call rate ≥ 95%, MAF ≥ 0.05, and HWE *p* > 1 × 10^−6^ filters to the merged dataset) was used for all ADMIXTURE and PCA analyses. Using the unrelated, LD-pruned dataset, we inferred individual ancestry with ADMIXTURE (v1.3) under unsupervised models for K = 1–5, using 10-fold cross-validation to select K by the minimum CV error [38,39]. The individual-by-cluster ancestry proportion matrices were visualised in R using pophelper for barplots [40]. Principal component analysis (PCA) [20,21] was run in PLINK on the same LD-pruned set. PCA plots were generated with ggplot2 v3.5.0 [22].

### 2.5. Genetic Differentiation

We estimated pairwise genetic differentiation as Weir & Cockerham’s $F_{ST}$ [23] for all breed pairs in PLINK (--fst --within) [14] and set negative estimates to zero, as they arise from sampling variance and do not indicate biological “negative differentiation”. Pairwise mean $F_{ST}$ values were assembled into a matrix and visualised as a heat map using the R package ggplot2 v3.5.0 [22].

### 2.6. Demographic History

We inferred recent historical effective population size ($N_{e}$) from linkage disequilibrium using SNeP v1.1 [18]. For each breed, analyses were run on the QC-filtered autosomal dataset (35,857 SNPs; no additional pruning or relatedness filtering beyond QC), estimating pairwise r^2^ in 50-kb bins across 50 kb–4 Mb with a minimum r^2^ of 0.01. We fitted the Sved–Feldman expectation [41] with mutation correction α = 2 [42] under a recombination rate of 1 × 10^−8^ bp per generation to obtain $N_{e}$ trajectories. Curves are interpreted only over distance ranges with adequate SNP support, acknowledging the sensitivity of LD-based $N_{e}$ to sample size and array density [43,44].

### 2.7. Selective-Sweep Detection and Functional Annotation of Candidate Regions

Per-breed datasets were derived by subsetting the QC-filtered binary PLINK files (call rate ≥ 95%, MAF ≥ 0.05, HWE *p* > 1 × 10^−6^; 35,857 SNPs retained) to breed-specific sample lists, and chromosomes were split to facilitate downstream processing. Variant Call Format (VCF) files were exported per chromosome, normalised with BCFtools v1.9 [45] to split multiallelic sites and remove duplicate records, restricted to biallelic SNPs, then block-gzipped and indexed. Phasing was performed with Beagle v5.1 [46]. These steps ensured a consistent, phased, autosomal, biallelic SNP panel for all breeds suitable for haplotype-based statistics.

Per-statistic computations

We combined five complementary statistics per breed for DCMS:

Integrated Haplotype Score (iHS): Computed within population using Selscan v.2.1 [47,48] ingesting phased VCFs by chromosome with no ancestral polarisation (polarize_vcf = FALSE). Extended haplotype homozygosity was scanned with an integration cap of 500 kb (maxgap = $1\times 10^{5}$). Genome-wide iHS values were derived with ihh2ihs function (min MAF = 0.05; frequency bins = 0.1) and transformed to $\left|iHS\right|$ for DCMS.

Number of Segregating Sites by Length (nSL): The nSL was computed with Selscan v.2.1 [48] on phased VCFs for each chromosome. Raw nSL Scores were normalised within derived allele-frequency bins (0.05 to 0.95 in 0.05 increments) by converting to per-bin Z-scores; if a bin contained fewer than a dynamic minimum of points (≥max (30, 2% of sites)), global Z-normalisation was used for those entries.

Haplotype homozygosity (H12): Computed following the implementation of [49] using 25-SNP sliding windows with a 1-SNP step; windows with <3 observed haplotypes were discarded. The H12 values were assigned to window centres for aggregation.

Pooled heterozygosity ($ZH_{p}$): Within fixed 500 kb windows on a shared genome-wide grid (anchored at base 1), we summed the major-allele count ($n_{maj}$) and minor-allele counts ($n_{min}$) per SNP from breed-specific allele-count files and computed pooled heterozygosity per window as $H_{p}=\frac{2\sum n_{maj}\sum n_{min}}{{\left(\sum n_{maj+}\sum n_{min}\right)}^{2}}$, following ([50]). Windows with <10 SNPs were excluded. $H_{p}$ was Z-standardised within breed ($ZH_{p}$); low $ZH_{p}$ indicates local dips in diversity.

Tajima’s D: Computed in VCFtools v0.1.15 [51] in fixed, non-overlapping 500 kb windows per chromosome, retaining windows with ≥5 SNPs.

All five statistics were aggregated to a common set of non-overlapping 500 kb windows per autosome. For iHS, nSL, and H12, we averaged all values whose genomic positions fell within a window; $ZH_{p}$ and Tajima’s D already matched the window grid.

### 2.8. Empirical p-Values, De-Correlation, and DCMS

Within each breed, window-level values were converted to empirical, rank-based *p*-values using the fractional-rank approach implemented in R package MINOTAUR [52], with tail directions defined a priori: right-tailed for |iHS| and H12, two-tailed for nSL, left-tailed for ZHp and Tajima’s D. To account for redundancy among statistics, we estimated a robust covariance matrix (M-estimator) and computed de-correlated composite of multiple signals (DCMS) scores by summing logit-transformed *p*-values with correlation-aware weights as described by [11] and implemented in MINOTAUR [52]. We converted DCMS scores to upper-tail *p*-values by robust normal modelling of the genome-wide score distribution and controlled FDR at 5% [53]. Windows with q < 0.05 were declared DCMS outliers within breed.

### 2.9. Functional Annotation of Candidate Regions

Significant DCMS windows were queried against the Ensembl (*Capra hircus*, ARS1) via Representational State Transfer (REST) application programming interface [54]. Gene symbols and annotations were harmonised using BiomaRt v2.60.1 [55].

## 3. Results

### 3.1. Genomic Patterns of Inbreeding and Runs of Homozygosity in Indian Salem Black and Global Goat Breeds

Table 1 summarizes observed heterozygosity (Ho, in %), multiple genomic inbreeding coefficients (in %), ROH-based metrics by length class, and total ROH genome coverage (in Mb) for six goat populations representing Indian Salem Black and global breeds. Observed heterozygosity (Ho) varied significantly (*p* = 1.12 × 10^−44^), among populations, ranging from 9.60 ± 3.59% in IN_SAL to 39.48 ± 0.88% in TZ_TOG. Within the global group, NG_RSK also showed high heterozygosity (38.56 ± 0.92%), whereas the remaining breeds (MG_DIA, OM_JAK, PK_KAC) exhibited intermediate levels (32.54–33.33%). Analyses of total ROH length and genome coverage indicated that IN_SAL had the largest ROH coverage (980.08 ± 322.46 Mb), followed by PK_KAC (468.66 ± 38.97 Mb) and NG_RSK (414.64 ± 32.07 Mb), whereas TZ_TOG and MG_DIA had the lowest ROH totals (219.26 ± 38.01 Mb and 306.21 ± 52.73 Mb, respectively). Notably, IN_SAL was the only breed showing a complete absence of ROH in the 1–4 Mb length class, indicating no detectable ROH in this length class under the current detection parameters. This may reflect a genuine absence of ancient inbreeding signal, though it should be interpreted cautiously given that ROH detection in short segments is sensitive to SNP density, genotyping platform, and sample size [31,32]. A striking proportion of IN_SAL’s ROH consisted of segments over 8 Mb, contributing to its elevated $F_{ROH}$ > 8Mb (38.03%). By contrast, TZ_TOG exhibited the lowest $F_{ROH}$ > 8Mb (4.56%). The overall ROH-based inbreeding coefficient ($F_{ROH}$) followed the same pattern, being highest in IN_SAL (0.409 ± 0.135), lowest in TZ_TOG and MG_DIA (0.091 ± 0.015 and 0.098 ± 0.026, respectively). Negative or near-zero inbreeding estimates were common in multiple metrics outside $F_{ROH}$. Notably, $F_{GRM}$ estimates were negative in most global populations, ranging from −11.47% to −0.58%. In contrast, IN_SAL consistently displayed positive values across all homozygosity-based coefficients ($F_{GRM}$ 46.94 ± 40.62%, $F_{HOM}$ 46.94 ± 21.27% and $F_{UNI}$ 46.94 ± 9.77%), reinforcing the signal of substantial recent inbreeding.

### 3.2. Correlation Analysis of Multiple Measures of Genomic Inbreeding Metrics in Salem Black and Global Goat Breeds

**Figure 1:**
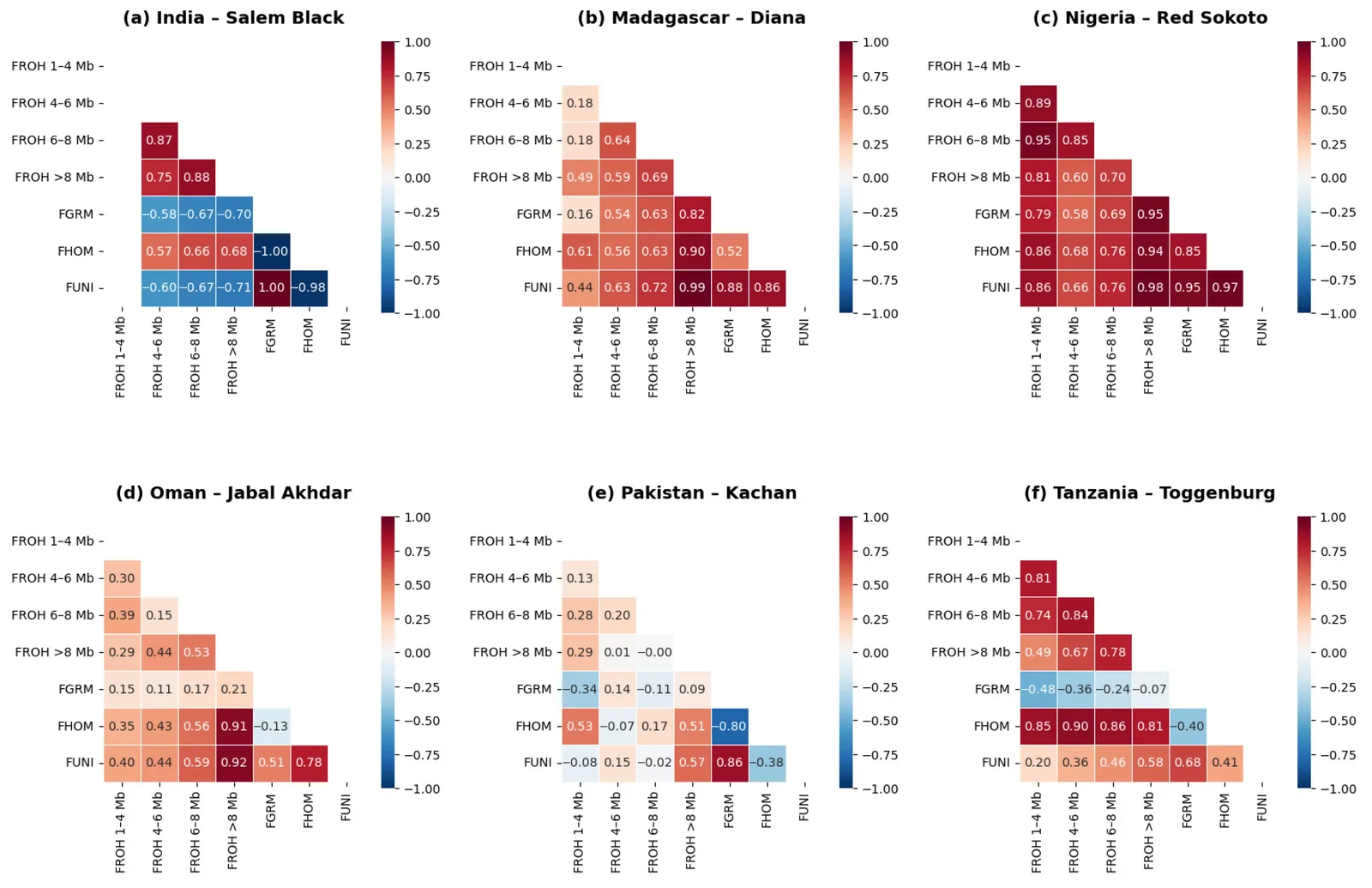
Pearson correlation matrices for inbreeding metrics and ROH categories in Salem Black and global goat breeds: Panel (**a**) show the Indian Salem Black. Panels (**b**–**f**) shows the global goat breeds: Madagascar–Diana (**b**), Nigeria–Red Sokoto (**c**), Oman–Jabal Akhdar (**d**), Pakistan–Kachan (**e**), and Tanzania–Toggenburg (**f**). The matrices display correlations between different inbreeding metrics, including ROH-based inbreeding coefficients ($F_{ROH}$) for segment lengths (1–4 Mb, 4–6 Mb, 6–8 Mb, >8 Mb), genetic relationship matrix-based inbreeding coefficient ($F_{GRM}$), inbreeding coefficient based on excess homozygosity ($F_{HOM}$), and inbreeding coefficient from the correlation between uniting gametes ($F_{UNI}$).

Figure 1 presents the correlation matrices among multiple genomic inbreeding coefficients and ROH length classes in six goat populations. In the IN_SAL population, longer ROH classes ($F_{ROH}$ 6–8 and $F_{ROH}$ > 8) exhibited strong positive intercorrelations (r = 0.75–0.88; *p* < 0.001) and moderate positive correlations with $F_{HOM}$: r = 0.57; *p* < 0.001, but strong negative correlations with $F_{GRM}$ and $F_{UNI}$ (r = −0.58 and −0.60; *p* < 0.001). Notably, $F_{HOM}$ and $F_{GRM}$ were perfectly negatively correlated (r = −1.00; *p* < 0.001), whereas $F_{GRM}$ and $F_{UNI}$ showed perfect positive correlation (r = 1.00; *p* < 0.001), highlighting their opposing sensitivities to homozygosity versus population-specific allele frequencies. The MG_DIA population showed low-to-strong positive correlations across most ROH length classes (r = 0.18–0.99; *p* < 0.05–0.001). Longer ROHs (>8 Mb) were strongly positively associated with $F_{UNI}$ (r = 0.99; *p* < 0.001), $F_{HOM}$ (r = 0.90; *p* < 0.001), and $F_{GRM}$ (r = 0.82; *p* < 0.001), indicating that recent autozygosity dominates in this population. In the NG_RSK population, correlations were uniformly strong and positive (r = 0.60–0.99; *p* < 0.001). The OM_JAK population displayed moderate correlations among ROH classes (r = 0.29–0.39; *p* < 0.05–0.001), with stronger associations between $F_{ROH}$ > 8 and $F_{ROH}$ (r = 0.92; *p* < 0.001). In the PK_KAC population, correlations among ROH classes were generally low to moderate (r = 0.13 to 0.29). However, $F_{GRM}$ was strongly positively correlated with $F_{UNI}$ (r = 0.86; *p* < 0.001) but strongly negatively correlated with $F_{HOM}$ (r = −0.80; *p* < 0.001), pointing to substantial allele frequency variation and limited linkage between ROH-based and homozygosity-based inbreeding metrics, consistent with low recent inbreeding. The TZ_TOG population exhibited strong positive correlations among ROH length classes (r = 0.49–0.84; *p* < 0.001), collectively indicating that recent inbreeding is the primary contributor to elevated genomic homozygosity in this breed.

### 3.3. Population Structure and Demographics

#### 3.3.1. ADMIXTURE Analysis

**Figure 2:**
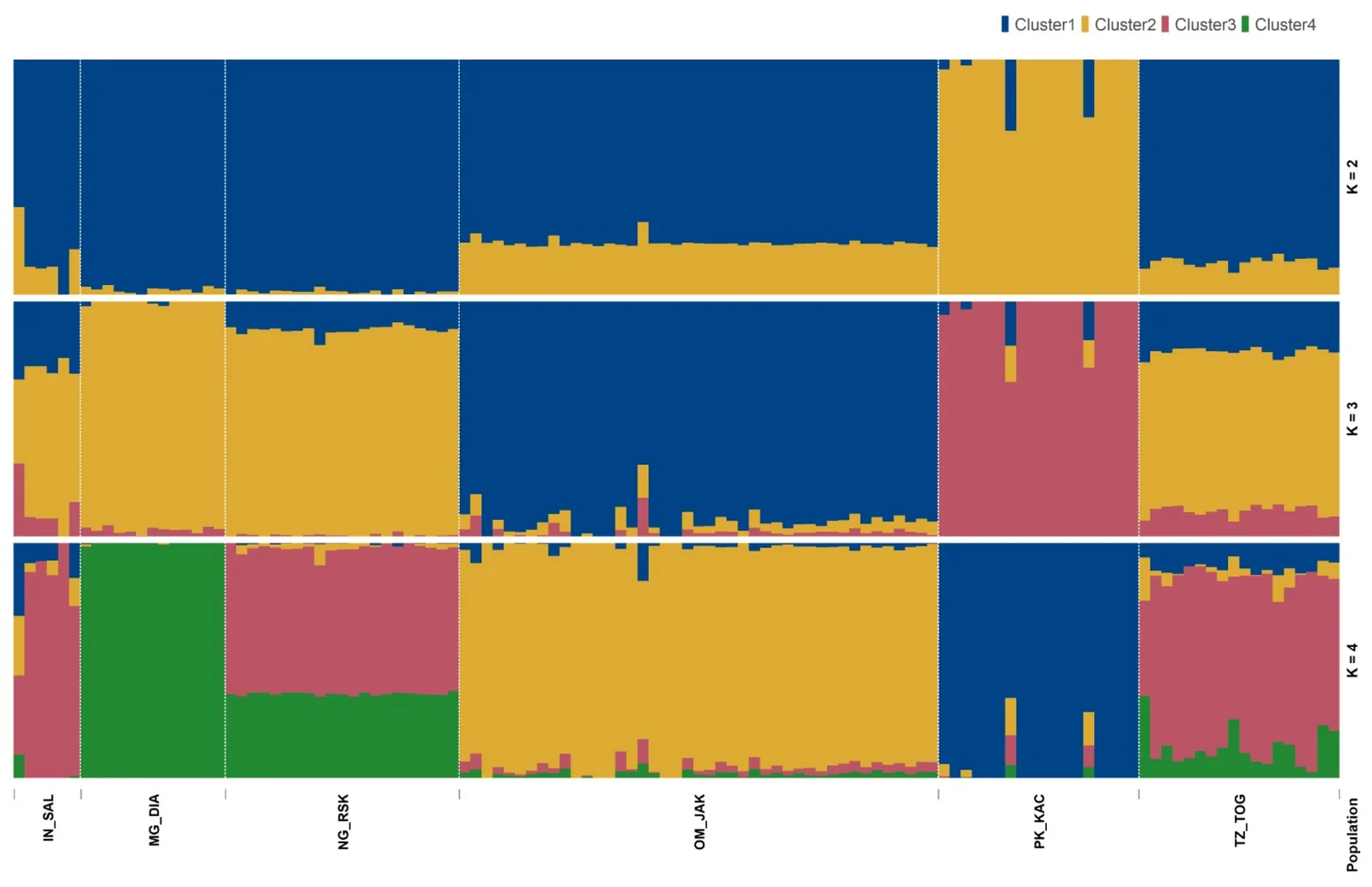
ADMIXTURE-inferred population structure of Salem Black and global goat breeds. Each individual (vertical bar) is partitioned into coloured segments representing membership coefficients in K ancestral clusters estimated with ADMIXTURE for K = 1–5 (Figure S1). Populations are ordered by country and ecotype: IN_SAL = India–Salem Black; MG_DIA = Madagascar–Diana; NG_RSK = Nigeria–Red Sokoto; OM_JAK = Oman–Jabal Akhdar; PK_KAC = Pakistan–Kachan; TZ_TOG = Tanzania–Toggenburg goat (Outgroup). Dashed white lines separate populations; cluster colours are consistent across K.

ADMIXTURE cross-validation supported a model with four ancestry components (K=4). The cross-validation error declined from 0.7664 at K=1 to 0.7270, 0.6976 and 0.6842 at K = 2–4, reached a minimum of 0.6842 at K=4 and then increased to 0.6987 at K=5 (Figure S1). We therefore focused on K=4 as the most informative cluster for interpretation of population structure (Figure 2). The genetic structure of the six goat populations was assessed using a model-based clustering approach with ancestral populations ranging from K=2−4 (Figure 2). Clear population substructures and varying levels of admixture were evident. At K=2, the 120 unrelated goats formed two major ancestries, with IN_SAL, MG_DIA, and NG_RSK clustering together, while OM_JAK, PK_KAC, and TZ_TOG formed the second group. K=3 revealed three ancestral groups with moderate admixture in PK_KAC and TZ_TOG, while OM_JAK remained genetically consistent, and MG_DIA began to diverge from the others. At K=4, IN_SAL was dominated by Cluster 1 with minor Cluster 3 influence; MG_DIA showed an almost exclusive Cluster 4 background; NG_RSK displayed mixed ancestry from Clusters 3 and 4; OM_JAK remained largely pure for Cluster 2; PK_KAC showed substantial admixture involving Clusters 1, 3, and 2; and TZ_TOG exhibited high admixture with near-equal contributions from Clusters 3 and 4. PK_KAC, TZ_TOG, and NG_RSK showed the strongest signatures of admixture, while MG_DIA and OM_JAK appeared genetically distinct with minimal external gene flow.

#### 3.3.2. Genome-Wide SNP Variation Using Principal Component Analysis

**Figure 3:**
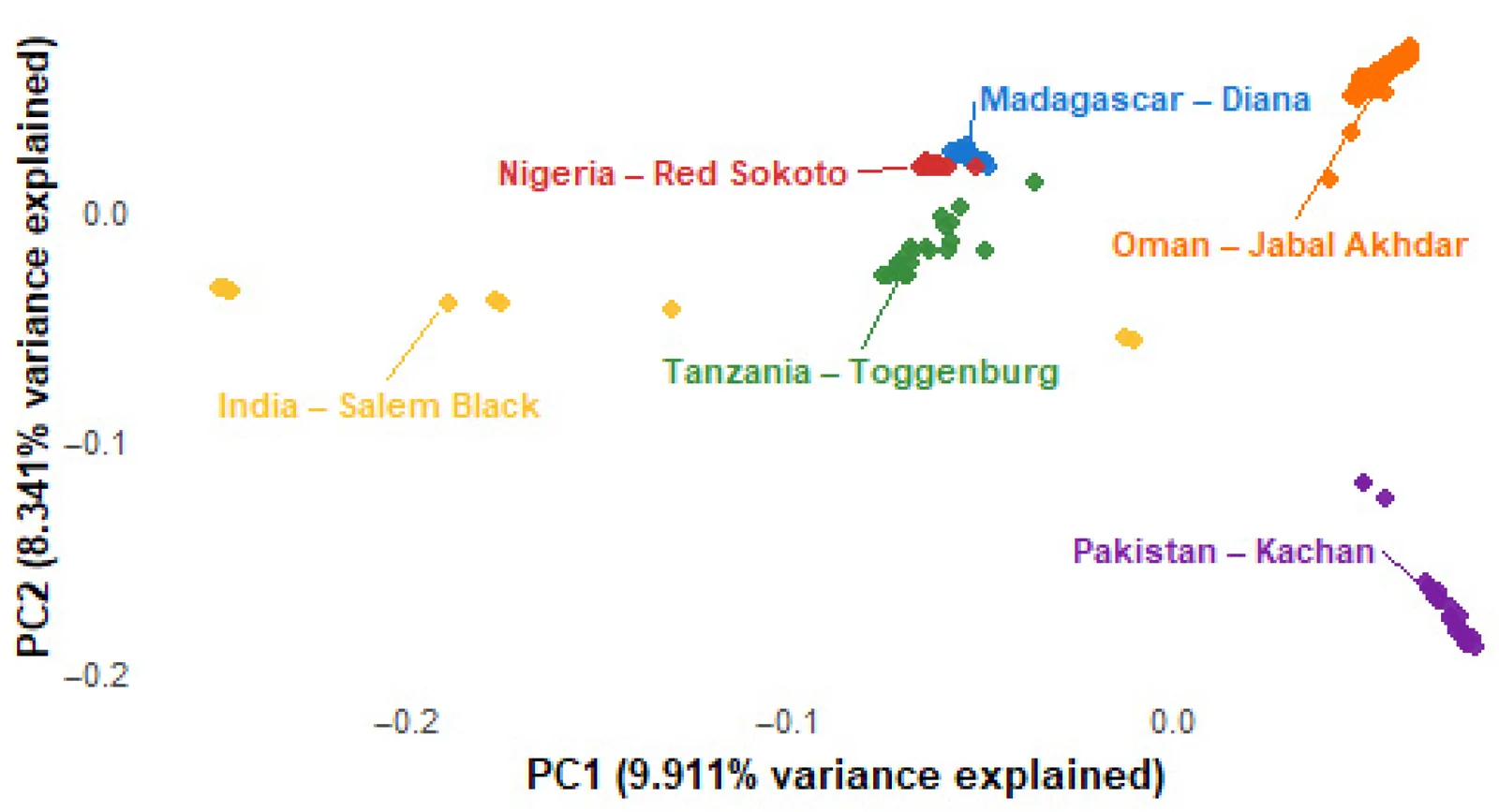
Principal component analysis of genome-wide SNP variation in Salem Black and global goat breeds. IN_SAL = India–Salem Black; MG_DIA = Madagascar–Diana; NG_RSK = Nigeria–Red Sokoto; OM_JAK = Oman–Jabal Akhdar; PK_KAC = Pakistan–Kachan; TZ_TOG = Tanzania–Toggenburg goat breeds (Outgroup). PC=Principal component. Total PC = 18.3% (Table S1).

Principal Component Analysis (PCA) was conducted to assess and visualise the genome-wide SNP variation in IN_SAL and global goat populations (Figure 3). PC1 explained 9.91% of the total variance, while PC2 accounted for 8.34%, together capturing 18.3% of the genomic variation as detailed in Supplementary Table S1. PC1 separated the OM_JAK population along the positive axis from the IN_SAL population along the negative axis, representing the strongest axis of genetic differentiation. PC2 distinguished the PK_KAC population, which formed a discrete cluster on the negative side of PC2, indicating a distinct genetic background. A central cluster comprised the MG_DIA, NG_RSK, and TZ_TOG populations, reflecting significant overlap and close genetic affinity among African goat breeds despite their wide geographic distribution.

#### 3.3.3. Genome-Wide Pairwise Fst

**Figure 4:**
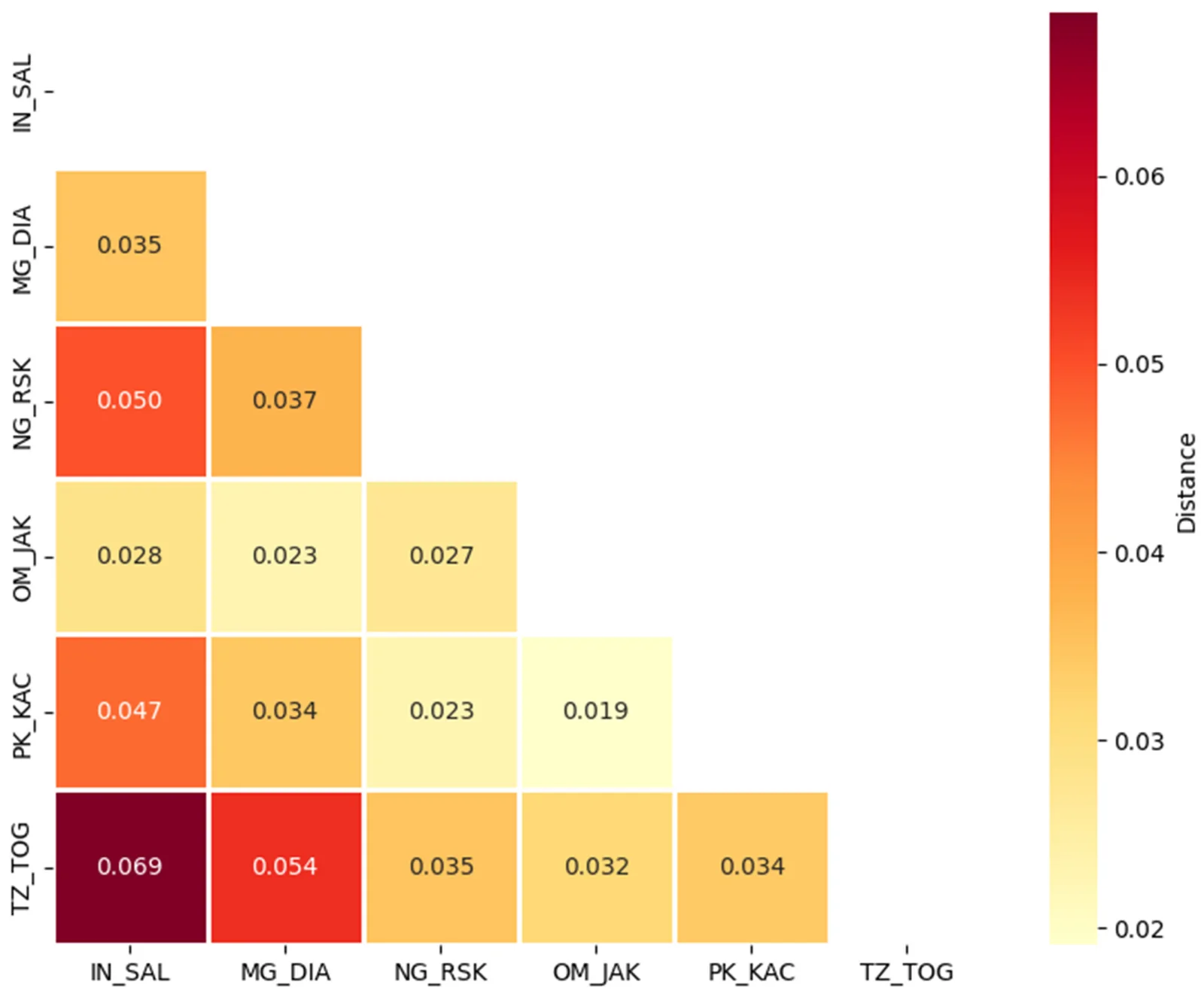
Heatmap showing genome-wide pairwise Weir–Cockerham FST between Indian Salem (IN_SAL = India–Salem Black) and global goat breeds (MG_DIA = Madagascar–Diana; NG_RSK = Nigeria–Red Sokoto; OM_JAK = Oman–Jabal Akhdar; PK_KAC = Pakistan–Kachan; TZ_TOG = Tanzania–Toggenburg).

Pairwise Weir–Cockerham $F_{ST}$ among the six goat breeds revealed varying levels of genetic differentiation ranged from 0.019 to 0.069 (Figure 4). The highest genetic differentiation in this study was observed between IN_SAL and TZ_TOG (outgroup); $F_{ST}=0.069$ indicating substantial divergence, consistent with long-term geographic isolation. Moderate genetic differentiation was found between IN_SAL and MG_DIA $F_{ST}$ 0.035, followed by NG_RSK $F_{ST}$ 0.050 and OM_JAK $F_{ST}$ 0.054. The lowest differentiation was observed between OM_JAK and PK_KAC $F_{ST}=0.019$. These patterns generally mirrored ADMIXTURE and PCA, highlighting lower genetic distance among ecologically similar breeds and higher divergence between populations adapted to contrasting environments or selected under distinct breeding objectives.

#### 3.3.4. Effective Population Size $N_{e}$ Trajectories

**Figure 5:**
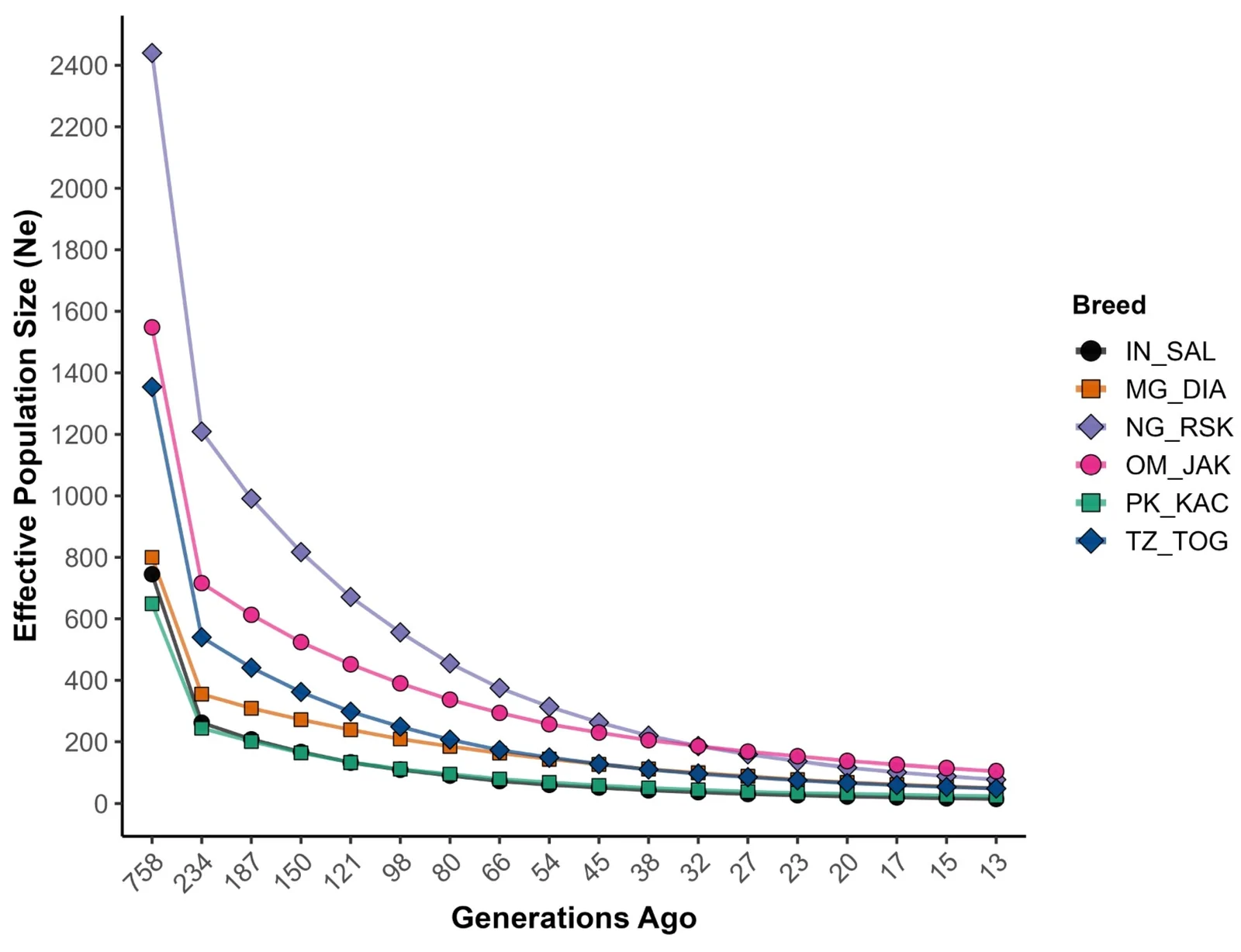
Historical trends in effective population size of Indian Salem (IN_SAL = India–Salem Black) and global goat breeds (MG_DIA = Madagascar–Diana; NG_RSK = Nigeria–Red Sokoto; OM_JAK = Oman–Jabal Akhdar; PK_KAC = Pakistan–Kachan; TZ_TOG = Tanzania–Toggenburg).

$N_{e}$ was reconstructed from 13 to 758 generations ago for six goat populations (Figure 5), revealing varying degrees of contraction or stability in each breed. At the deepest time point (758 generations ago), NG_RSK exhibited the highest $N_{e}$ which was above 2400, followed by OM_JAK (~1600), TZ_TOG (~1400), MG_DIA (~800), IN_SAL (~790) and PK_KAC (~700). By intermediate timescales (~121 generations ago), declines were pronounced, and overlap was observed in the $N_{e}$ of IN_SAL and PK_KAC (~190). Near the most recent time point (~13 generations ago), all breeds converged toward critically low Ne values (below 200), with IN_SAL reaching the lowest recent Ne of approximately 15 at the most recent time point (~13 generations ago). Because SNeP calculations can be sensitive to sample size and SNP density, these plots primarily provide a relative sense of demographic trends rather than absolute population sizes.

### 3.4. ROH Islands and De-Correlated Composite of Multiple Signals in India and Global Goat Breeds

#### 3.4.1. ROH Islands

**Figure 6:**
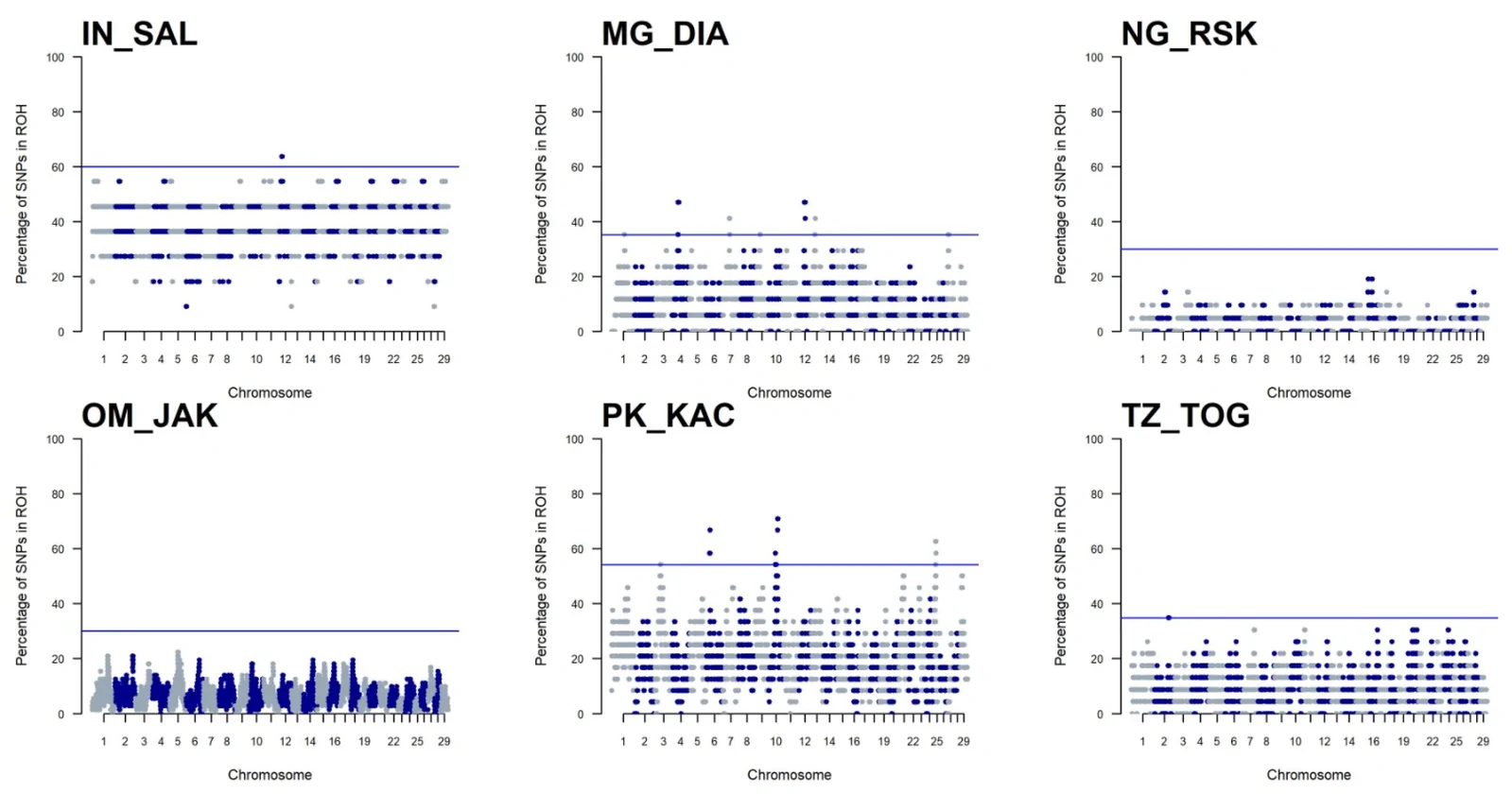
Runs of homozygosity islands in Salem Black and global goat breeds. Indian Salem Black (IN_SAL) and global goat populations (MG_DIA = Madagascar–Diana; NG_RSK = Nigeria–Red Sokoto; OM_JAK = Oman–Jabal Akhdar; PK_KAC = Pakistan–Kachan; TZ_TOG = Tanzania–Toggenburg).

Figure 6 illustrates the distribution of ROH islands across Indian Salem and global goat breeds, highlighting clear breed-specific variation. In the IN_SAL population, a single ROH island was detected on chromosome 12, present in 63.64% of the sampled individuals and encompassing 13 unique genes (Table S2). The MG_DIA breed exhibited multiple ROH islands distributed across chromosomes 1, 4, 7, 9, and 27, with frequencies ranging from 35.29% to 47.06% of individuals, collectively harboring 110 unique genes (Table S2). In the PK_KAC population, ROH islands were identified on chromosomes 3, 6, 10, and 25, occurring in 54.17%, 60.41%, 61.67%, and 58.34% of individuals, respectively, and containing a total of 109 unique genes (Table S2). The TZ_TOG breed displayed a single ROH island on chromosome 2, detected in 34.78% of individuals and comprising 23 unique genes (Table S2). No ROH islands were detected in NG_RSK or OM_JAK, likely due to their higher heterozygosity and moderate inbreeding levels (Ho = 38.56% and $F_{ROH}$ = 0.119, respectively), which reduce the probability of any genomic region reaching consistent autozygosity across individuals.

#### 3.4.2. De-Correlated Composite of Multiple Signals (DCMS)

**Figure 7:**
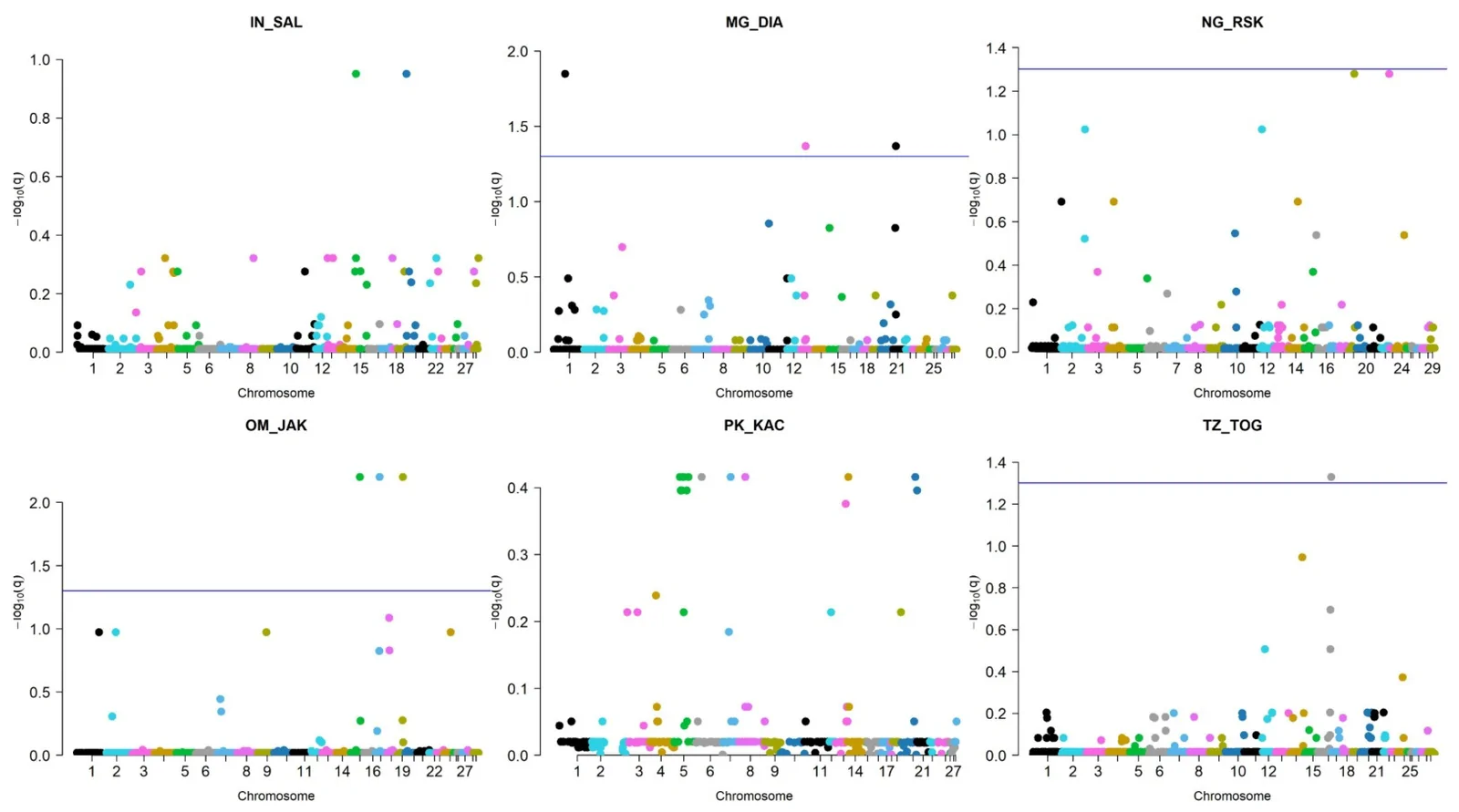
Breed-wise DCMS scans were carried out on fixed 500-kb windows, with significance defined at a 5% genome-wide false-discovery rate (q < 0.05); the reference line in each panel marks this threshold. Indian Salem Black (IN_SAL) and global goat populations (MG_DIA = Madagascar–Diana; NG_RSK = Nigeria–Red Sokoto; OM_JAK = Oman–Jabal Akhdar; PK_KAC = Pakistan–Kachan; TZ_TOG = Tanzania–Toggenburg). Dots of different colours represent different chromosomes.

Figure 7 presents breed-level DCMS scans, performed using fixed 500-kb sliding windows with a genome-wide significance threshold of 5% false discovery rate (q < 0.05). The reference line in each panel indicates this significance cutoff. A total of 7 windows surpassed the significance threshold across the six breeds (detailed in Table S3), distributed as follows: IN_SAL = 0, MG_DIA = 3, NG_RSK = 0, OM_JAK = 3, PK_KAC = 0 and TZ_TOG = 1. Annotation of genes within these significant windows revealed 38 protein-coding genes with valid symbols (Table S4). MG_DIA revealed 13 genes on chromosome 1 (*NEPRO*, *BOC*, *SPICE1*, *SIDT1*), chromosome 13 (*FAM107B*, *CDNF*, *HSPA14*, *SNORD22*, *SUV39H2*, *DCLRE1C*, *MEIG1*, *ACBD7*), and chromosome 21 (*NTRK3*). OM_JAK had 18 genes on chromosome 15 (*GRIK4*, *ARHGEF12*, *TLCD5*, *POU2F3*) and chromosome 19 (*PSME3*, *AOC2*, *G6PC1*, *AARSD1*, *PTGES3L*, *RUNDC1*, *RPL27*, *IFI35*, *VAT1*, *RND2*, *BRCA1*, *NBR1*, *TMEM106A*, *ARL4D*). TZ_TOG had 8 genes on chromosome 16 (*RPS6KC1*, *ANGEL2*, *VASH2*, *FLVCR1*, *SPATA45*, *TATDN3*, *NSL1* and *BATF3*). Detailed window coordinates and DCMS statistics are provided in Tables S3 and S4.

## 4. Discussion

This study used comprehensive genome-wide analyses to examine genetic diversity, inbreeding, population structure and adaptive evolution in indigenous goat populations raised mainly under smallholder and agro-pastoral systems. These production systems underpin rural livelihoods across much of the developing world and exert strong environmental and demographic pressures that shape livestock genomes. By integrating runs of homozygosity, multiple genomic inbreeding estimators, demographic reconstruction, and composite selection scans, we reveal how historical isolation, breeding practices, and ecological constraints interact to influence genomic architecture. Our results demonstrate a striking contrast between the extreme autozygosity observed in Salem Black goats and the adaptive genomic signatures detected in African, South Asian, and Middle Eastern populations, highlighting how genetic diversity underpins resilience and long-term sustainability. These findings fill critical knowledge gaps regarding the balance between inbreeding and adaptation in indigenous goats and provide a genomic framework to inform conservation planning and breeding strategies in Salem Black goats.

### 4.1. Genomic Patterns of Inbreeding and Runs of Homozygosity in Salem Black and Global Goat Breeds

Our genomic analysis of runs of homozygosity (ROH) and inbreeding coefficients across six goat populations provides valuable insights into their genetic structure, influenced by historical bottlenecks and local breeding practices (Table 1). The IN_SAL breed showed the strongest signals of recent intense inbreeding, with markedly low observed heterozygosity ($H_{O}$ = 9.6%), extensive ROH coverage (980.08 Mb total; $F_{ROH}$ = 0.409), and a high proportion of long ROH (>8 Mb; 38.03% of the genome), a finding that warrants immediate attention for conservation. These genetic signatures indicate autozygosity from close mating, as described by [16], and suggest severe genetic diversity loss, likely due to prolonged isolation in semi-arid regions. The observed extended ROH in IN_SAL is common among some Indian goat breeds under a small-holder production system, such as Jharkhand Black, Marwari, Surti, and Assam Hill [9]. Ref. [9] reported varying inbreeding levels in Indian goats based on genome-wide ROH signatures ($F_{ROH}$ from 0.004 in Changthangi to 0.602 in Jharkhand Black). Higher ROH counts and genome coverage have been noted in Indian meat breeds, speculatively tied to prolonged isolation and intense selection [9,56]. The longer ROH observed here echo findings in several Indian meat breeds (Jharkhand Black, Marwari, Surti, and Assam Hill), signaling recent inbreeding from management practices and selective pressures. The high inbreeding reported in IN_SAL was similar to that of the IBE (Siberian Ibex) population, a wild goat population, which had the highest inbreeding coefficient (0.4780) and showed an exceptionally high total ROH length (11,788.44 Mb), indicating a higher level of inbreeding within this population [57]. This was consistent with the findings of previous studies, where the modern wild bezoar from Iran displayed the highest F_ROH_ [58]. The low observed heterozygosity suggests compromised genetic diversity in IN_SAL, which may affect long-term fitness and adaptive potential, which contrasts with the moderate diversity reported in other Indian breeds, such as Attapaddy and Malabari goats ($H_{O}$ = ≈0.3755; [10]). The global goat populations had observed heterozygosity which ranged from 32.54 in MG_DIA to 39.48% in TZ_TOG, which implies that the populations are in good genetic health and sustained diversity under smallholder systems that encourage natural outcrossing and minimal artificial selection [59]. High values recorded for genomic measures ($F_{\_HOM}$ ≈ 0.469, $F_{\_UNI}$ ≈ 0.469, $F_{\_GRM}$ ≈ 0.469) in IN_SAL, contrast the reported low to moderate estimates in different species under different production systems [17,27]. Speculatively, such extreme homozygosity raises risks of inbreeding depression, including reduced fertility and increased vulnerability to deleterious alleles, as reflected in elevated homozygosity-based coefficients. The critically low genetic diversity and small effective population size observed in IN_SAL highlight the need for structured conservation strategies, including controlled introduction of genetic material from related breeds and implementation of directional mating programs, as demonstrated in similar recovery efforts for the Gembrong goat breed in Indonesia [60]. These findings may serve as a genomic baseline to inform conservation planning under existing national frameworks such as the All India Coordinated Research Project on Goat Improvement (AICRP-Goat), which is well positioned to incorporate genomic tools alongside traditional breed management practices for IN_SAL recovery.

### 4.2. Correlation Analysis of Multiple Measures of Genomic Inbreeding Metrics in Salem Black and Global Goat Populations

Our analysis in Salem Black and global goat populations detailed the associations between various genomic inbreeding metrics, providing essential insights into the genetic structure of these populations (Figure 1). This study highlights the critical role of different inbreeding coefficients in assessing the genetic makeup and historical breeding practices of goats under smallholder production levels. Moderate to strong correlations observed between $F_{ROH}$, $F_{GRM}$, $F_{HOM}$ and $F_{UNI}$ across all breeds indicate their effectiveness in capturing homozygosity, which correlates with inbreeding levels that have accumulated over generations. Ref. [61] differentiated the implications of short and long ROH, noting that shorter ROH segments generally indicate ancient inbreeding, while longer segments reflect more recent inbreeding events. This understanding is crucial as it elucidates how different lengths of ROH can provide insights into the temporal dynamics of inbreeding within these populations. The consistently positive correlation between $F_{ROH}$ and $F_{HOM}$ across all breeds [61] reflects the sensitivity of both metrics to recent autozygosity. This metric’s ability to signal the status of genetic health in a population is valuable for identifying genetic resilience and potential areas for genetic conservation or enhancement within breeding programs. The negative correlation between $F_{HOM}$ and $F_{GRM}$, especially noted in breeds like the IN_SAL, TZ_TOG, PK_KAC and OM_JAK, underscores the contrasting genetic insights these metrics provide. While $F_{HOM}$ tends to indicate increased homozygosity, $F_{GRM}$ identifies regions of genetic heterogeneity, suggesting that these breeds retain significant genetic diversity despite historical inbreeding [61]. This divergence is crucial for maintaining breed health and adaptability, as it suggests that pockets of genetic diversity exist which can be critical for long-term breed sustainability. These correlation trends offer valuable insights into how different inbreeding metrics can be interpreted in the context of breed management. They highlight the importance of using a combination of these metrics to gain a comprehensive understanding of both inbreeding levels and the broader genetic health of the breeds, which is crucial for making informed decisions in breeding programs aimed at maintaining genetic diversity and vigor [17].

### 4.3. Genetic Relationships and Ancestry Patterns

Earlier microsatellite work by [62] reported high within-population gene diversity as compared to European and Asian goat breeds. Our Admixture, PCA and $F_{ST}$ results are consistent with the Indian Ocean trade corridor hypothesis, which facilitated livestock exchange, gene flow, and dispersal of goat germplasm [28]. The Indian Ocean and Sudan Sahelian trade corridor genomic background defined by IN_SAL, MG_DIA, NG_RSK, OM_JAK, PK_KAC and TZ_TOG is characterised by shared ancestry (cluster 3), low to moderate pairwise $F_{ST}$ within the group (0.028–0.069) and tight PCA clustering among the Sahelian corridors (MG_DIA, NG_RSK and TZ_TOG). The overlapping PCA position between IN_SAL and goats from Sahelian corridors (NG_RSK, MG_DIA and TZ_TOG) is an indication of shared ancestry. This broad division is consistent with the well-documented split between South-West Asian and African goat lineages following post-domestication dispersal from the Fertile Crescent [63,64]. Madagascar Diana (MG_DIA) exhibited near-exclusive assignment to Cluster 4 (>95%), underscoring its strong East African maternal heritage introduced by Bantu-speaking migrations, with minimal subsequent admixture despite the island’s complex settlement history [65,66]. NG_RSK, PK_KAC and TZ_TOG displayed the highest levels of admixture. NG_RSK showed balanced contributions from Cluster 4, reflecting gene flow within West and Central African pastoral networks [67,68]. PK_KAC exhibited the most complex ancestry, with substantial proportions of Clusters 1, 2, and 3, likely resulting from historical interactions along ancient trade routes connecting the Indus Valley, Persia, and the Arabian Peninsula [69,70]. Finally, TZ_TOG was highly admixed in Cluster 4, a pattern expected from its documented origin as a composite of introduced Tanzania Toggenburg germplasm crossed with local East African goats since the early 20th century [71,72]. Collectively, the admixture, Fst and PCA patterns elucidate the interplay of migration, admixture, and selection in shaping goat genetic architecture, with implications for breed conservation and targeted improvement. The distinct positioning of Asian and Middle East populations (OM_JAK, IN_SAL and PK_KAC) versus the African core cluster emphasizes the need for region-specific breeding strategies to harness unique alleles, such as those conferring altitude tolerance in OM_JAK or prolificacy in IN_SAL, while leveraging the African cluster’s diversity for crossbreeding to enhance productivity under climate variability [59,73].

### 4.4. Demographic Contraction

The observed demographic contraction in IN_SAL is particularly noteworthy when interpreted alongside earlier microsatellite-based studies. Previous work in Salem Black goats reported moderate to high polymorphism information content (PIC > 0.25) and positive deviations from the Hardy–Weinberg equilibrium in a large proportion of loci [74], indicating historically maintained genetic variability. Comparable patterns have been documented in other Indian goat breeds [56,62,75], suggesting that many indigenous populations retained substantial diversity prior to recent demographic pressures. The sharp reduction in contemporary *N*_e_ inferred from LD patterns, therefore likely reflects recent demographic and mating structure changes rather than an abrupt loss of heterozygosity. Similar convergence toward low contemporary *N*_e_ has been widely reported in global goat populations using LD-based methods [28,76], reinforcing the interpretation that modern breeding and management practices are major drivers of recent genetic erosion. This scenario is characteristic of populations experiencing an increase in inbreeding due to reduced effective breeders, non-random mating, and limited gene flow, where erosion of adaptive variation may precede detectable losses in neutral diversity. Consequently, the immediate concern for IN_SAL is the gradual accumulation of inbreeding and the potential onset of inbreeding depression, particularly for fitness-related and adaptive traits, rather than sudden genetic collapse. It is important to acknowledge that LD-based *N*_e_ estimates derived from SNeP are sensitive to factors such as sample size, SNP density, allele frequency distribution, and assumptions regarding recombination rates. As such, these estimates should be interpreted primarily as relative indicators of demographic trends rather than precise absolute population sizes. Nonetheless, the concordant decline observed across geographically and genetically distinct populations strengthens confidence in the inferred demographic patterns. Collectively, these trajectories highlight shared historical bottlenecks likely shaped by a combination of human-mediated selection, migration, breed formation, and more recent intensification of farming systems. For geographically isolated or locally adapted populations such as IN_SAL, MG_DIA and PK_KAC these findings reinforce the urgency of implementing structured breeding and conservation programs. The exceptionally high ancestral *N*_e_ observed in NG_RSK (>2400; Figure 5) suggests a historically large and genetically connected population, consistent with extensive pastoral production systems due to the ECOWAS treaty that allows transboundary movement of livestock in Sahel-Sudanian corridors [77]. Strategies aimed at maintaining *N*_e_, optimizing mating designs, and facilitating controlled gene flow will be critical to preserving long-term adaptability and resilience under changing production and climatic conditions. The small retained sample size for IN_SAL (*n* = 7 post-kinship filtering) should be noted as a limitation. LD-based Ne estimation is sensitive to sample size and may overestimate the severity of recent demographic decline [43,44], while ROH detection power for short segments is reduced in small samples. These findings should therefore be interpreted as indicative of severe genomic erosion rather than precise absolute estimates.

### 4.5. ROH Islands and De-Correlated Composite of Multiple Signals (DCMS) Reveal Potential Candidate Genes Related to Breed Characteristics in Salem Black and Global Goat Populations

#### 4.5.1. Candidate Genes Within ROH Islands

The ROH island analyses elucidate the complex genetic landscape of Salem Black and global goat populations, emphasizing the influence of historical and contemporary selection pressures in defining breed-specific traits. This genetic overview, illustrated in Figure 6 and elaborated in Supplementary Table S2, traces the evolutionary paths shaped by varied environmental and human-related factors. For the IN_SAL and PK_KAC breeds, significant ROH islands highlight regions of intense historical selection likely spurred by environmental challenges, as documented by [16]. In contrast, the MG_DIA and TZ_TOG breeds exhibit fewer ROH islands, suggesting a history of milder selection pressures. This could indicate a broader genetic diversity within these breeds, possibly due to extensive mating pools common in pastoral and agropastoral systems [37,47,78]. In the PK_KAC breed, genes located within ROH islands like *BMPR1B*, *PDHA2*, *UNC5C*, *FBN1*, *CYP19* and *RORA* have been associated with fecundity and litter size in different species [79]. The PK_KAC adaptation catalogue includes *PDLIM5*, *RAP1GDS1*, *STPG2*, and *TSPAN5*, and is primarily linked to cellular structure, signaling, or neural/sperm tail functions. Another noteworthy candidate gene, *CYP19*, is associated with spermatozoa concentration and motility in humans, with polymorphisms of *CYP19* resulting in reduced sperm concentration within normozoospermic males [80]. In the IN_SAL, *LCP1* correlates with immune infiltration [81]. *RCBTB2* is considered a candidate tumor suppressor gene that may exhibit loss or low expression in prostate cancer (PCa). Its functions in inhibiting cancer and promoting cilia formation position it as a viable therapeutic target [82]. *FNDC3A* is required for adhesion between spermatids and Sertoli cells [83]. Ref. [84] reported that the *RB1* gene has been mapped in goats and is located on goat chromosome 12. The protein produced by the *RB1* gene (pRB) is a central regulator of the cell cycle, preventing cells from dividing uncontrollably. The functions of *ITM2B* have long been focused on regulating neurological dysfunction [85].

In MG_DIA breed the ROH islands contained functionally coherent gene sets:

- Innate immunity (*DEFB* family). Beta-defensin genes encode antimicrobial peptides critical for pathogen resistance. Selection here likely reflects historical pressure from endemic diseases and provides pre-adaptation to increased pathogen loads expected with warmer, wetter climates [61].
- Metabolic Efficiency and Production (*CD36*, *PITX1*, *GPRC6A*). These genes regulate lipid metabolism, nutrient sensing, and mammary development, supporting energy thriftiness, fat utilization during scarcity, and balanced milk/meat production in low-input systems [86,87].
- Reproductive Fitness and Plasticity (*CCDC39*, *SOX2*, *FXR1*). Involved in flagellar function, developmental plasticity, and stress-responsive translation, these promote fertility, optimal litter size, and rapid adaptation to environmental cues [88,89].
- Developmental Robustness (*SMAD5*, *TGFBI*): Part of TGF-β signaling, contributing to skeletal and tissue resilience suited to harsh terrains [59].

The clustering of these functionally related genes within ROH islands suggests a polygenic adaptive pattern involving immune function, resource-use efficiency, and reproductive traits that may contribute to survival and productivity in marginal agro-ecosystems. These genomic features position MG_DIA as a resilient breed amid climate-driven challenges like drought, forage variability, heat stress, and emerging diseases. The absence of ROH islands in NG_RSK and OM_JAK likely reflects high within-population diversity and moderate inbreeding rather than a lack of adaptive potential, as selection in these breeds may act through polygenic or soft-sweep mechanisms not readily captured by ROH incidence approaches [11,12].

In TZ_TOG, metabolic genes dominate the identified ROH islands, suggesting selection for energy efficiency and nutrient utilization in Tanzanian Toggenburg goats, which often face feed scarcity and heat stress. *IDH1* encodes isocitrate dehydrogenase 1, a key enzyme in the tricarboxylic acid (TCA) cycle that catalyzes the oxidative decarboxylation of isocitrate to alpha-ketoglutarate, generating *NADPH* for cellular redox balance. Similarly, *ACADL*, involved in long-chain fatty acid beta-oxidation, supports lipid metabolism critical for milk fat synthesis in dairy breeds. *CPS1*, a urea cycle enzyme, facilitates ammonia detoxification, which may enhance resilience to high-protein diets or metabolic stress in tropical climates. *RPE* encodes ribulose-5-phosphate-3-epimerase, part of the pentose phosphate pathway, contributing to nucleotide synthesis and oxidative stress response. *MDH1B*, a putative malate dehydrogenase, likely aids in cytosolic malate-aspartate shuttling for energy production. These metabolic adaptations align with broader patterns in African dairy goats, where ROH hotspots often overlap genes for thermotolerance and feed efficiency [90]. Transcriptional and regulatory genes within ROH islands point to fine-tuned gene expression networks under selection. *KLF7*, a Kruppel-like transcription factor, modulates insulin sensitivity in beta cells and skeletal muscle while inhibiting adipogenesis, potentially optimizing energy partitioning for lactation over fat storage in dairy animals. *CREB1*, responsive to cAMP signaling, regulates nutrient metabolism and has been linked to growth and stress responses in livestock. *METTL21A*, a methyltransferase near *CREB1*, may influence epigenetic modifications, while *KANSL1L* contributes to histone acetylation complexes, affecting chromatin accessibility. These regulators could drive adaptive gene expression in response to environmental cues, consistent with ROH analyses in other goat breeds showing selection on transcriptional hubs for domestication and production traits [57,91]. Notably, a cluster of gamma-crystallin genes (*CRYGA*, *CRYGB*, *CRYGC*, *CRYGD*) resides in ROH islands, traditionally known for maintaining lens transparency as structural eye lens proteins. Mutations in these genes cause cataracts in humans and mice, but their presence in goat ROH suggests non-ocular roles, such as stress chaperones or osmotic regulators, as observed in other species. In arid Tanzanian conditions, improved vision or UV protection could be advantageous, though linkage disequilibrium with nearby genes like *C2H2orf80* (a putative zinc finger protein) might explain their fixation [90,92].

#### 4.5.2. Candidate Genes Within DCMS Windows

Annotation of the significant genomic windows across the three goat populations revealed breed-specific patterns of genes associated with environmental adaptation, stress physiology, metabolic regulation, and reproductive performance. In MG_DIA goats, several candidate genes within significant DCMS windows (e.g., *HSPA14*, *SUV39H2*, *DCLRE1C*) are functionally associated with heat-stress response, chromatin modification, and DNA repair, suggesting these regions may reflect long-term adaptation to the humid and warm environments of Madagascar. The presence of *MEIG1* and *ACBD7* further points toward selection on reproductive efficiency and lipid metabolism [90]. The OM_JAK population showed the highest number of annotated genes, consistent with stronger selection pressure in the arid, mountainous ecology of Oman. Candidate genes such as *BRCA1*, *PSME3*, *IFI35*, and *G6PC1* are potentially associated with pathways involved in immune surveillance, metabolic homeostasis, oxidative stress management, and cellular repair, though causal roles require experimental validation [29,93]. These indicate that survival in harsh, nutrient-limited habitats has favored goats with efficient immune function and robust DNA-repair mechanisms. For TZ_TOG goats, candidate genes related to angiogenesis (*VASH2*), heme transport (*FLVCR1*), and immune regulation (*BATF3*) were identified within the significant DCMS window, suggesting possible roles in oxygen transport, thermoregulation, and host defense in East African tropical environments [94]. These findings align with the climatic conditions in East African tropical systems, where heat load and vector-borne diseases impose continuous selective pressure. Overall, the gene sets identified across the three breeds reflect distinct evolutionary trajectories shaped by local ecological conditions. The presence of heat-shock, immunological, metabolic, and DNA-repair candidate genes across breeds is consistent with the hypothesis that adaptive selection in goats may be influenced by climatic stress, pathogen exposure, and reproductive demands, consistent with previous reports in caprine genomic studies.

### 4.6. Methodological Limitations and Next Steps

This study combined whole-genome resequencing of IN_SAL with 50K SNP-array genotypes for the five global comparison breeds, introducing SNP ascertainment bias: the array was designed from discovery panels that did not include Salem Black, so the shared SNP set may under-represent variation private to IN_SAL and over-represent variation common to the array’s design breeds. This can inflate apparent homozygosity in IN_SAL and bias diversity/differentiation comparisons toward regions best captured by the array. LD-based Ne estimates from SNeP should likewise be read comparatively rather than as precise census correlates [95], given their sensitivity to sample size and marker density, particularly for the small IN_SAL sample. Similarly, DCMS combines iHS, nSL, H12, ZHp, and Tajima’s D into one composite score, but modest population sizes limit power to resolve closely linked signals. DCMS windows should be treated as prioritised candidates for replication, not confirmed adaptive loci [11,95].

The absence of DCMS outliers in IN_SAL likely reflects reduced power from extreme inbreeding rather than a true absence of selection. Haplotype-based statistics detect selection by contrasting a region against the genomic background—a contrast that becomes uninformative when homozygosity is uniformly elevated, compressing the score distribution below the FDR threshold. This is a recognised limitation of composite scans in highly inbred populations [11,12]. The chromosome-12 ROH island, present in 63.64% of IN_SAL individuals, offers independent evidence of localised selection that ROH detected but DCMS did not.

Expanding IN_SAL sampling across its home tract in Tamil Nadu via denser genotyping or additional resequencing would reduce this platform mismatch. Pairing that with phenotype and management metadata would let candidate genes be tested for genotype–phenotype associations. Extending comparisons to other Indian goat breeds would also clarify whether IN_SAL’s genomic erosion is breed-specific or part of a broader pattern.

## 5. Conclusions

The present study represents the first whole-genome investigation of the Salem Black goat, an economically important but understudied Indian breed. Our results demonstrate that Salem Black has undergone severe recent inbreeding, resulting in critically low genetic diversity and an alarmingly small effective population size. These genomic patterns signal high risk of inbreeding depression and loss of adaptive potential, underscoring the urgent need for conservation interventions such as controlled introgression from related breeds and establishment of structured breeding programs. Comparative analyses confirmed the genetic distinctiveness of Salem Black within global goat diversity and revealed clear signatures of positive selection in three other indigenous populations adapted to harsh environments. Genes related to heat tolerance, immune response, DNA repair, and reproductive efficiency were prominently enriched, reinforcing the role of natural and human-mediated selection in shaping climate-resilient traits in tropical goats. The absence of detectable selection signatures in Salem Black itself likely reflects the overwhelming impact of genome-wide homozygosity rather than a true lack of adaptive evolution. To address this critical situation, emergency conservation measures are warranted for IN_SAL, including: (i) cryopreservation of semen and embryos from genetically distinct individuals; (ii) genomic relationship matrix-guided mating schemes to restrict matings between close relatives; and (iii) managed genetic introgression from PK_KAC, the genomically closest breed, to restore heterozygosity without compromising breed identity. These measures could be coordinated through the AICRP-Goat framework with periodic genomic monitoring to track diversity recovery. These findings provide a genomic baseline for future breeding and conservation strategies, emphasize the value of indigenous goat genetic resources for climate-resilient livestock production, and highlight the power of whole-genome approaches to guide sustainable management of smallholder goat populations in developing countries.

## Figures and Tables

**Table 1:** Analysis of runs of homozygosity (ROH) and inbreeding coefficients in Indian Salem Black and global goat breeds.

Metric	IN_SAL	MG_DIA	NG_RSK	OM_JAK	PK_KAC	TZ_TOG	*p*-Value
Ho (%)	9.60 ^c^ ± 3.59	32.54 ^b^ ± 1.01	38.56 ^a^ ± 0.92	33.13 ^b^ ± 1.09	33.33 ^b^ ± 1.03	39.48 ^a^ ± 0.88	1.12 × 10^−44^
*F*_GRM_ (%)	46.94 ^a^ ± 40.62	−7.72 ^bc^ ± 3.00	−11.47 ^c^ ± 3.18	−3.76 ^bc^ ± 3.32	−9.21 ^c^ ± 3.56	−0.58 ^b^ ± 3.49	1.47 × 10^−3^
*F*_HOM_ (%)	46.94 ^a^ ± 21.27	2.58 ^bc^ ± 2.77	6.55 ^b^ ± 2.52	10.34 ^b^ ± 2.31	−3.03 ^d^ ± 2.47	0.06 ^c^ ± 2.81	3.36 × 10^−8^
*F*_UNI_ (%)	46.94 ^a^ ± 9.77	2.58 ^c^ ± 2.52	6.69 ^bc^ ± 2.37	10.08 ^b^ ± 2.18	−3.40 ^e^ ± 2.39	0.03 ^d^ ± 1.75	5.94 × 10^−25^
*F*_ROH_ (Mean ± SE)	0.409 ^a^ ± 0.135	0.098 ^d^ ± 0.026	0.141 ^c^ ± 0.017	0.119 ^cd^ ± 0.011	0.195 ^b^ ± 0.016	0.091 ^d^ ± 0.015	1.62 × 10^−13^
*F*_ROH_ 1–4 Mb (Mean ± SE)	ND	0.017 ^ab^ ± 0.001	0.019 ^a^ ± 0.001	0.016 ^b^ ± 0.001	0.014 ^b^ ± 0.001	0.018 ^ab^ ± 0.002	1.41 × 10^−19^
*F*_ROH_ 4–6 Mb (Mean ± SE)	0.008 ^d^ ± 0.003	0.013 ^c^ ± 0.001	0.015 ^ab^ ± 0.001	0.014 ^b^ ± 0.001	0.019 ^a^ ± 0.001	0.014 ^b^ ± 0.002	2.27 × 10^−14^
*F*_ROH_ 6–8 Mb (Mean ± SE)	0.020 ^a^ ± 0.007	0.014 ^bc^ ± 0.001	0.011 ^d^ ± 0.001	0.012 ^c^ ± 0.001	0.018 ^b^ ± 0.001	0.012 ^c^ ± 0.002	8.55 × 10^−12^
*F*_ROH_ > 8 Mb (Mean ± SE)	0.380 ^a^ ± 0.125	0.053 ^d^ ± 0.026	0.094 ^c^ ± 0.016	0.076 ^cd^ ± 0.011	0.143 ^b^ ± 0.015	0.045 ^d^± 0.010	1.30 × 10^−14^
ROH genome coverage (Mb)	980.08 ^a^ ± 322.46	306.21 ^d^ ± 52.73	414.64 ^c^ ± 32.07	324.09 ^cd^ ± 26.27	468.66 ^b^ ± 38.97	219.26 ^e^ ± 38.01	1.62 × 10^−13^

Different letters (a–e) within a row denote statistically significant differences (p < 0.05) after one-way ANOVA followed by Tukey–Kramer post-hoc tests. Sample sizes used for all metrics: IN_SAL (*n* = 7), MG_DIA (*n* = 17), NG_RSK (*n* = 21), OM_JAK (*n* = 72), PK_KAC (*n* = 24), TZ_TOG (*n* = 23). No ROH segments in the 1–4 Mb class were detected in IN_SAL (Salem Black); the corresponding FROH 1–4 Mb value is therefore reported as ND (not detected) instead of a numerical value. Note that the relatively small sample size for IN_SAL limits the precision of parameter estimates and should be interpreted with caution. Ho: observed heterozygosity; ROH Genome Coverage: length of autosomes covered by ROH segments in each breed; $F_{GRM}$: variance-standardized genomic relationship inbreeding coefficient, reflecting overall allele sharing; $F_{HOM}$: homozygosity-based inbreeding coefficient exceeding Hardy–Weinberg expectations; $F_{UNI}$: inbreeding coefficient from the correlation between uniting gametes; $F_{ROH}$: overall inbreeding coefficient estimated as the proportion of the autosomal genome covered by ROH of any length; $F_{ROH}$ 1–4 Mb, $F_{ROH}$ 4–6 Mb, $F_{ROH}$ 6–8 Mb, $F_{ROH}$ > 8 Mb: proportion of the autosomal genome in ROH within each length class, broadly reflecting ancient, intermediate, recent, and very recent inbreeding, respectively; ROH genome coverage: total length (Mb) of autosomal segments in ROH per individual; IN_SAL = India–Salem Black; MG_DIA = Madagascar–Diana; NG_RSK = Nigeria–Red Sokoto; OM_JAK = Oman–Jabal Akhdar; PK_KAC = Pakistan–Kachan; TZ_TOG = Tanzania–Toggenburg.

## Data Availability

The sequencing data generated in this study are deposited in NCBI under BioProject PRJNA1302180 (BioSamples SAMN50465512–SAMN50465522; SRA SRR34954288–SRR34954298). Additional analysis files are available from the corresponding author upon reasonable request.

## Supplementary Materials

The following supporting information can be downloaded at https://www.mdpi.com/article/10.3390/ani16193123/s1. Figure S1: Cross-validation error plot of India Salem and global goat populations. Table S1: Mean estimates and Proportion of variance contributed by genome-wide SNP in India Salem Black and global goat population. Table S2: Number and Symbols of Genes Identified within ROH Islands in Indian Salem Black and Global Goat Breeds. Table S3: DCMS-significant genomic windows (FDR < 5%) identified in Indian Salem Black and global goat populations. Table S4: Candidate genes identified within DCMS-significant windows in Indian Salem Black and global goat populations. Table S5: Per-sample sequencing statistics (raw data volume, Q30 scores, GC content, and mapping rates). Table S6: Complete SNP filtering cascade and final analysis subsets for the merged Salem Black WGS and AdaptMap array dataset.
