# Supplementary material for: Genomic Insights into Inbreeding, Population Structure, and Selection Signatures in Salem Black and Global Goat Breeds
Source: Animals (Basel). 2026 Oct 5;16(19):3123. doi: 10.3390/ani16193123 (PMC13663511; doi:10.3390/ani16193123)
Supplement: Supplementary file 1 [file animals-16-03123-s001.zip › Table S1.pdf]

Table S1: Mean estimates and Proportion of variance contributed by genome-wide SNP in India Salem Black and global goat population.

Population	mean_PC1	mean_PC2	prop_PC1	prop_PC2
Nigeria – Red Sokoto	-0.06258726	0.01833021	7.781057	0.9524283
Tanzania – Toggenburg	-0.06512483	-0.01842157	8.424805	0.9619457
Madagascar – Diana	-0.05366377	0.02280987	5.720435	1.4748324
India – Salem Black	-0.17685649	-0.04157011	62.131054	4.8984601
Oman – Jabal Akhdar	0.05523841	0.05823227	6.061066	9.6122408
Pakistan – Kachan	0.07053098	-0.17018577	9.881584	82.1000928

Prop=Proportion; PC=Principal component
