# Supplementary material for: Genomic Insights into Inbreeding, Population Structure, and Selection Signatures in Salem Black and Global Goat Breeds
Source: Animals (Basel). 2026 Oct 5;16(19):3123. doi: 10.3390/ani16193123 (PMC13663511; doi:10.3390/ani16193123)
Supplement: Supplementary file 1 [file animals-16-03123-s001.zip › Table S2 ROH Island.pdf]

Table S2. Number and Symbols of Genes Identified within ROH Islands in Indian Salem Black and Global Goat Breeds

Breed	Chromosome	Number of Genes	Gene Symbols
PK_KAC	3	2	*NTNG1, PRMT6*
	6	7	*BMPR1B, PDHA2, PDLIM5, RAP1GDS1, STPG2, TSPAN5, UNC5C*
	10	82	*ADAM10, ALDH1A2, ANXA2, AP4E1, AQP9, ARPP19, ATP8B4, BCL2L10, BNIP2, C10H15orf65, C2CD4B, CCNB2, CCPG1, CEP152, CGNL1, COPS2, CTXN2, CYP19A1, DMXL2, DNAAF4, DTWD1, DUT, EID1, FAM214A, FAM227B, FAM81A, FBN1, FGF7, FOXB1, GABPB1, GALK2, GCNT3, GLDN, GNB5, GTF2A2, HDC, ICE2, LEO1, LIPC, LYSMD2, MAPK6, MINDY2, MNS1, MYEF2, MYO1E, MYO5A, MYO5C, MYZAP, NEDD4, ONECUT1, PIGB, POLR2M, PRTG, PYGO1, RAB27A, RFX7, RNF111, RORA, RSL24D1, SCG3, SECISBP2L, SEMA6D, SHC4, SLC12A1, SLC24A5, SLC27A2, SLTM, SPPL2A, TCF12, TEX9, TLN2, TMOD2, TMOD3, TNFAIP8L3, TPM1, TRPM7, UNC13C, USP50, USP8, VPS13C, WDR72, ZNF280D*
	25	18	*ABCC1, ABCC6, BFAR, C25H16orf45, CPPED1, ERCC4, FOPNL, MARF1, MKL2, MPV17L, MYH11, NDE1, NTAN1, PARN, PDXDC1, RRN3, SHISA9, SNX29*
TZ_TOG	2	23	*DYTN, MDH1B, FASTKD2, CPO, KLF7, CREB1, METTL21A, CCNYL1, FZD5, PLEKHM3, CRYGD, CRYGC, CRYGB, CRYGA, C2H2orf80, IDH1, PIKFYVE, PTH2R, MAP2, UNC80, RPE, KANSL1L, ACADL, MYL1, LANCL1* and *CPS1*.
IN_SAL	12	13	*ZC3H13, CPB2, LCP1, LRRC63, LRCH1, ESD, SUCLA2, NUDT15, ITM2B, RB1, FNDC3A, MIR2285O-1, RCBTB2*
MG_DIA	1	5	*CCDC39, DNAJC19, FXR1, SOX2, TTC14*
	27	22	*AGA, ASB5, DEFB, DEFB1, DEFB10, DEFB108B, DEFB13, DEFB4A, DEFB7, EBD, GPM6A, LAP, MIR2887-2, NEIL3, RN18S1, SPAG11B, SPATA4, SPCS3, TAP, VEGFC, WDR17, ZNF705A*

	4	26	*ARMC10, CACNA2D1, CCDC146, CD36, DNAJC2, FAM185A, FBXL13, FGL2, GNAI1, GNAT3, GSAP, HGF, LHFPL3, LRRC17, MAGI2, NAPEPLD, ORC5, PHTF2, PMPCB, PSMC2, PTPN12, RELN, RSBN1L, SEMA3C, SLC26A5, TMEM60*
	7	32	*C7H5orf15, C7H5orf24, CAMLG, CATSPER3, CDKL3, CDKN2AIPNL, CXCL14, DDX46, FBXL21, FSTL4, H2AFY, IL9, JADE2, LECT2, NEUROG1, PCBD2, PITX1, PPP2CA, SAR1B, SEC24A, SKP1, SLC25A48, SMAD5, SMIM32, SPOCK1, TCF7, TGFBI, TIFAB, TRPC7, TXNDC15, UBE2B, VDAC1*
	9	25	*CALHM4, CALHM5, CALHM6, COL10A1, DCBLD1, DSE, FAM162B, FRK, GOPC, GPRC6A, KPNA5, NEPN, NT5DC1, NUS1, RFX6, ROS1, RSPH4A, RWDD1, SLC35F1, SULT1C4, TRAPPC3L, TSPYL1, TSPYL4, VGLL2, ZUP1*
