# Supplementary material for: Genomic Insights into Inbreeding, Population Structure, and Selection Signatures in Salem Black and Global Goat Breeds
Source: Animals (Basel). 2026 Oct 5;16(19):3123. doi: 10.3390/ani16193123 (PMC13663511; doi:10.3390/ani16193123)
Supplement: Supplementary file 1 [file animals-16-03123-s001.zip › Table S6.pdf]

Table S6. Complete SNP filtering cascade and final analysis subsets for the merged Salem Black WGS and AdaptMap array dataset.

Stage	Dataset / Purpose	SNPs Before	SNPs Removed	SNPs Retained	Procedure / Criterion
1	Original AdaptMap array dataset (all breeds)	—	—	53,347	Autosomal SNPs (chromosomes 1–29) from the ADAPTmap genotyping array (CaprineSNP50 BeadChip)
2	Selected AdaptMap individuals (five comparative breeds)	53,347	0	53,347	--keep (OM_JAK n=72, MG_DIA n=17, NG_RSK n=21, TZ_TOG n=23, PK_KAC n=24); breed renaming and allele coding
3	Salem Black WGS (11 samples) — IN_SAL call set	—	—	40,376	GATK HaplotypeCaller v4.4.2.1 + ANGSD v0.918; per-sample filters: QD >2, missingness <0.1, MAF >0.05; autosomal biallelic SNPs only
4	Cross-dataset SNP harmonization (merge IN_SAL + AdaptMap)	40,376 + 53,347	3,290	37,086	--bmerge; extract common SNP IDs; allele and strand harmonization; 168 animals total (11 IN_SAL + 157 public)
5	Integrated dataset (all 168 samples, pre-QC)	37,086	0	37,086	Common autosomal SNP panel across all six breeds; no additional filter applied at this step
6	Integrated dataset after joint QC	37,086	1,229	35,857	Call rate ≥95%, MAF ≥0.05, HWE exact P $\geq 1\times10^{-6}$; individuals with >10% missing genotypes removed (none failed); this is the primary analysis set
7	Relatedness filtering	35,857 (markers) 168 (individuals)	—	35,857 (markers) 120 (individuals)	PI_HAT >0.25 pairs pruned per breed; 48 individuals removed; marker count unchanged
8	LD pruning for PCA and ADMIXTURE	35,857	12,218	23,639	PLINK --indep-pairwise 50 5 0.2 (50-SNP window, 5-SNP step, $r^2 \leq 0.2$); LD-pruned set used exclusively for PCA and ADMIXTURE
9	Final analysis subsets	—	—	35,857 / 23,639	Unpruned 35,857-SNP set: ROH, genomic inbreeding coefficients (FROH, FGRM, FHOM, FUNI), FST, SNeP Ne trajectories, DCMS selection scans LD-pruned 23,639-SNP set: PCA and ADMIXTURE only

-The 40,376 SNPs are the high-quality autosomal SNPs retained from the 11 Salem Black WGS samples after variant calling and sample-level filters. This figure is not the final analysis set; after cross-dataset harmonization and joint QC the primary analysis set comprises 35,857 SNPs.

a AdaptMap genotyping used the Illumina GoatSNP50 BeadChip (53,347 autosomal SNPs after excluding sex chromosomes and unplaced contigs).

b Salem Black WGS variant calling was performed with GATK HaplotypeCaller v4.4.2.1 and ANGSD v0.918 against the goat reference genome ARS1.2 (GCF_001704415.2) using BWA v0.7.17. Per-sample autosomal variant counts before final QC are shown in the per-sample table below.

c Cross-dataset harmonization: SNPs present in both the Salem Black call set and the AdaptMap panel were extracted; alleles were matched and strand-ambiguous SNPs (A/T, C/G) were removed.

d Joint QC filters applied to the merged dataset: genotype call rate ≥95% per SNP, minor allele frequency (MAF) ≥0.05, Hardy–Weinberg equilibrium exact test P $\geq 1\times10^{-6}$ (per breed). No individual failed the >10% per-individual missingness threshold.

e Relatedness filtering: pairs of individuals with PI_HAT >0.25 were identified within each breed; one individual per pair (with higher missingness) was removed. The final unrelated dataset comprised 120 individuals: IN_SAL n=7, OM_JAK n=43, MG_DIA n=13, NG_RSK n=21, TZ_TOG n=18, PK_KAC n=18.

f LD pruning parameters: PLINK v1.9 --indep-pairwise 50 5 0.2 (50-SNP sliding window, 5-SNP step, r^2 threshold 0.2). The LD-pruned 23,639-SNP set was used exclusively for PCA and ADMIXTURE; all other analyses used the full unpruned 35,857-SNP set.
