# Supplementary material for: Genomic Insights into Inbreeding, Population Structure, and Selection Signatures in Salem Black and Global Goat Breeds
Source: Animals (Basel). 2026 Oct 5;16(19):3123. doi: 10.3390/ani16193123 (PMC13663511; doi:10.3390/ani16193123)
Supplement: Supplementary file 1 [file animals-16-03123-s001.zip › Figure S1.pdf]

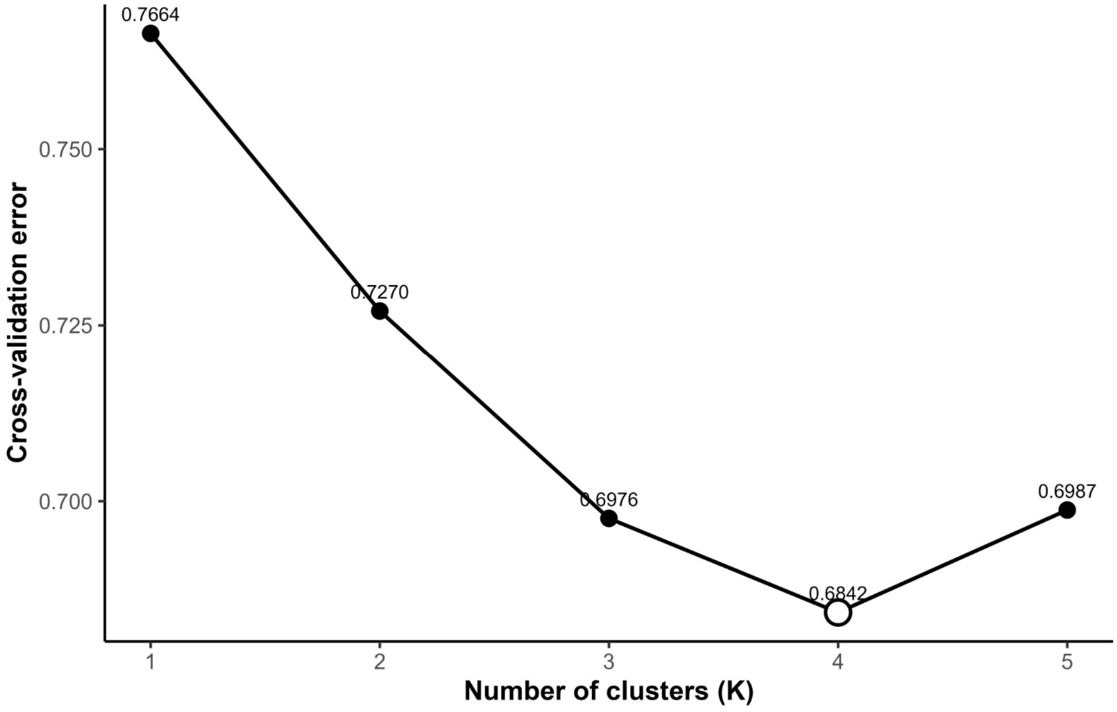


**Figure S1: Cross-validation error plot of India Salem and global goat populations**
